# Vasculogenic Potency of Bone Marrow- and Adipose Tissue-Derived Mesenchymal Stem/Stromal Cells Results in Differing Vascular Network Phenotypes in a Microfluidic Chip

**DOI:** 10.3389/fbioe.2022.764237

**Published:** 2022-02-08

**Authors:** Anastasiia Mykuliak, Alma Yrjänäinen, Antti-Juhana Mäki, Arjen Gebraad, Ella Lampela, Minna Kääriäinen, Toni-Karri Pakarinen, Pasi Kallio, Susanna Miettinen, Hanna Vuorenpää

**Affiliations:** ^1^ Adult Stem Cell Group, Faculty of Medicine and Health Technology, Tampere University, Tampere, Finland; ^2^ Research, Development and Innovation Centre, Tampere University Hospital, Tampere, Finland; ^3^ Micro- and Nanosystems Research Group, Faculty of Medicine and Health Technology, Tampere University, Tampere, Finland; ^4^ Department of Plastic and Reconstructive Surgery, Tampere University Hospital, Tampere, Finland; ^5^ Coxa Hospital for Joint Replacement, Tampere, Finland

**Keywords:** *in vitro* vascularization, mesenchymal stem cells, endothelial cells, organ-on-a-chip, pericytes, microfluidic chip

## Abstract

The vasculature is an essential, physiological element in virtually all human tissues. Formation of perfusable vasculature is therefore crucial for reliable tissue modeling. Three-dimensional vascular networks can be formed through the co-culture of endothelial cells (ECs) with stromal cells embedded in hydrogel. Mesenchymal stem/stromal cells (MSCs) derived from bone marrow (BMSCs) and adipose tissue (ASCs) are an attractive choice as stromal cells due to their natural perivascular localization and ability to support formation of mature and stable microvessels *in vitro*. So far, BMSCs and ASCs have been compared as vasculature-supporting cells in static cultures. In this study, BMSCs and ASCs were co-cultured with endothelial cells in a fibrin hydrogel in a perfusable microfluidic chip. We demonstrated that using MSCs of different origin resulted in vascular networks with distinct phenotypes. Both types of MSCs supported formation of mature and interconnected microvascular networks-on-a-chip. However, BMSCs induced formation of fully perfusable microvasculature with larger vessel area and length whereas ASCs resulted in partially perfusable microvascular networks. Immunostainings revealed that BMSCs outperformed ASCs in pericytic characteristics. Moreover, co-culture with BMSCs resulted in significantly higher expression levels of endothelial and pericyte-specific genes, as well as genes involved in vasculature maturation. Overall, our study provides valuable knowledge on the properties of MSCs as vasculature-supporting cells and highlights the importance of choosing the application-specific stromal cell source for vascularized organotypic models.

## Introduction

Microvascular networks are fundamental elements of almost every human tissue. The vascular system is responsible for the delivery of oxygen and nutrients and removal of waste products. The vasculature maintains tissue homeostasis and regeneration by providing angiocrine signaling and regulating the transport of bioactive compounds and cells. In addition, vasculature plays an important role in many pathological conditions ranging from inflammation to cancer (Augustin and Koh 2017). Thus, tissue vascularization is an essential element in establishing relevant microenvironments for *in vitro* tissue modeling. Despite great improvements in artificial vascularization achieved in recent years, generating mature and functional vascular structures *in vitro* remains one of the major challenges. Current approaches for *in vitro* vascularization mimic the process of *in vivo* vasculogenesis and angiogenesis by exploiting the intrinsic ability of ECs to self-organize into vessel-like structures and to create new vessels from pre-existing ones (Chen et al., 2013; Jeon et al., 2014; Jusoh et al., 2015; Guo et al., 2019; Li et al., 2019). While these approaches have proven successful for particular applications, most of them suffer from the lack of supportive mural cells that are fundamental elements of physiological vasculature (Sweeney and Foldes 2018). The aforementioned approaches often fail to reproduce the structure of the vessels thus leading to formation of immature vascular networks and require the use of multiple angiogenic growth factors to promote the neovessel formation and maintain stability of the vasculature (Whisler et al., 2014).

Direct co-culture of ECs with supporting stromal cells appears to be the most effective approach to recapitulate *in vivo* vascularization resulting in microvascular networks with physiologically relevant structure and functionality (Jeon et al., 2014; Li et al., 2017; Campisi et al., 2018; Paek et al., 2019). The stromal cells take on the role of perivascular cells that form the mural coat, provide the essential angiogenic growth factors, and stabilize the developing endothelial tubes (Stratman and Davis 2012; Sweeney and Foldes 2018). Human fibroblasts have been widely investigated as vasculature supporting cells (Li et al., 2017; Paek et al., 2019), however, adult mesenchymal stem cells (MSCs) have proven to induce formation of more mature (Grainger et al., 2013), stable (Rambøl et al., 2020), and less permeable microvessels (Grainger and Putnam 2011). Indeed, MSCs, such as well characterized and easily accessible bone marrow-derived mesenchymal stem/stromal cells (BMSCs) and adipose tissue-derived mesenchymal stem/stromal cells (ASCs), could serve as a promising vasculogenesis supporting cell source due to their natural perivascular localization (Lin et al., 2010).

Initially derived from the mesenchyme, BMSCs and ASCs share similar characteristics of cell surface expression patterns, differentiation potentials and expression of genes related to cell self-renewal (Dominici et al., 2006). In terms of pericytic properties, various studies have shown that MSC tissue source affects microvascular network development, maturation, and functionality (Verseijden et al., 2009; Grainger and Putnam 2011; Grainger et al., 2013; Pill et al., 2015; Pill et al., 2018). For instance, ASCs and BMSCs promote angiogenesis via distinct cytokine and protease expression mechanisms (Kachgal and Putnam 2011). Moreover, ASCs have been reported to support the formation of denser vascular network with significantly higher expression of vascular endothelial growth factor (VEGF) in ASC-EC co-culture compared to BMSC-EC co-culture (Pill et al., 2018). However, these studies were performed using static cultures. The present study is the first to compare the vasculogenic potency of BMSCs and ASCs in dynamic conditions.

The combination of three-dimensional (3D) culture with a microfluidic device has been utilized for the generation of human microvascular networks (Smith and Gerecht 2014) and state-of-the-art organ-on-a-chip models (Wu et al., 2020). The use of a microfluidic chip platform allows for precise and spatiotemporal control of culturing conditions as well as incorporating a fluid flow thus ensuring nutrients and oxygen supply, waste removal, and providing physiological mechanical stimuli (Zhang et al., 2021).

In the present study, we investigated the potential of BMSCs and ASCs to support vasculogenesis in a 3D microfluidic chip platform. We compared the capacity of BMSCs and ASCs to induce the formation of mature and robust microvascular networks by ECs and their function as perivascular cells. We particularly studied effect of MSCs on vessel characteristics such as vasculature area, average vessel diameter, vessel length, and vasculature perfusability. We assessed MSC distribution in terms of pericyte area and pericyte coverage by immunohistochemical staining and quantitative analysis. Furthermore, we evaluated the expression of main vasculogenesis related genes. We found that different types of MSCs support the formation of microvascular networks with distinct phenotypes and quality. While both MSCs induced the formation of lumenized and interconnected networks by ECs, BMSCs promoted the formation of more mature microvascular networks-on-a-chip than ASCs, leading to a higher degree of perfusability. The pericyte area and vasculature coverage by pericytes were significantly greater in EC-BMSC co-cultures compared to EC-ASC co-cultures. The gene expression analysis revealed significant differences in the expression of endothelial-specific and pericyte-specific genes, as well as genes involved in vasculature maturation. The results suggest that BMSCs have stronger vasculogenic potential in 3D microfluidic chip platform compared to ASCs. Our study provides valuable knowledge on the properties of MSCs as vasculature supporting cells that could be utilized in bioengineering and *in vitro* modeling applications to facilitate tissue vascularization.

## Materials and Methods

### Cell Isolation and Culture

Human BMSCs (herein denoted as BMSC 1–3) were isolated from bone marrow samples obtained from three donors (Supplementary Table S1) undergoing orthopedic surgery at the Tampere University Hospital Department of Orthopedics and Traumatology. Bone marrow samples were obtained with the donor’s written informed consent and processed under ethical approval of the Ethics Committee of the Expert Responsibility area of Tampere University Hospital, Tampere, Finland (R15174). The cells were isolated as described previously (Wang et al., 2019). The BMSCs were cultured in *α*-modified minimum essential medium (*α*-MEM; Gibco) supplemented with 5% human serum (HS; BioWest or Serana), 100 U/ml penicillin, 100 µg/ml streptomycin (Pen/Strep; Lonza), and 5 ng/ml basic fibroblast growth factor (bFGF; Miltenyi Biotec) and used between passages 3 and 6.

Human ASCs (herein denoted as ASC 1–3) were isolated from subcutaneous abdominal tissue samples obtained from three donors (Supplementary Table S1). Tissue samples were obtained at the Tampere University Hospital Department of Plastic Surgery with the donor’s written informed consent and processed under ethical approval of the Ethics Committee of the Expert Responsibility area of Tampere University Hospital (R15161). The cells were isolated as described previously (Kyllönen et al., 2013). The ASCs were cultured in *α*-MEM supplemented with 5% HS (BioWest, PAA or Serana), 100 U/ml penicillin, and 100 µg/ml streptomycin and used between passages 3 and 5. The mesenchymal origin of BMSCs and ASCs was confirmed by surface marker expression analysis with flow cytometry (Supplementary Table S2) and assessment of adipogenic and osteogenic differentiation potential (Supplementary Figure S1).

Pooled human umbilical vein endothelial cells (HUVECs) expressing green fluorescent protein (GFP) were commercially obtained from Cellworks. GFP-HUVECs were cultured in Endothelial Cell Growth Medium-2 Bullet Kit (EGM-2; Lonza) consisting of Endothelial Cell Growth Basal Medium (EBM-2) and Endothelial Cell Growth Medium-2 Supplements. Instead of the fetal bovine serum supplied with the Kit, 2% HS (BioWest) was used. The cells were used between passages 3 and 6.

### Microvascular Network Formation

Microvascular networks were formed inside microfluidic chips (DAX-1; Aim Biotech), consisting of a gel channel (1.3 mm wide and 250 µm high) flanked by two medium channels. Microvascular networks were formed through self-assembly of GFP-HUVECs and MSCs in fibrin hydrogel according to Jeon et al. (2014) with modifications. Briefly, GFP-HUVECs were combined with either BMSCs or ASCs at 5:1 cell ratio (final cell density 6 million cells/ml) and spun down. The cell pellet was then resuspended in 2 IU/ml human thrombin (Sigma-Aldrich, St. Louis, MI, United States) in EBM-2 and combined at 1:1 volume ratio with 5 mg/ml human fibrinogen (Sigma-Aldrich, St. Louis, MI, United States) in Dulbecco’s Phosphate Buffered Saline (DPBS; Lonza). The resulting mixture (10 µl) was quickly injected into the gel channel of the microfluidic chip and allowed to polymerize in a humidified chamber for 15–20 min at room temperature (RT). Microvascular networks were cultured in EGM-2 with 2% HS (BioWest). To enhance vascular network formation, interstitial flow was used throughout the culture period (Kim et al., 2016; Abe et al., 2019). The interstitial flow was generated by adding 90 µl of culture medium into both reservoirs of one medium channel and 50 µl of medium into both reservoirs of the other medium channel. This procedure was repeated daily when changing medium and the flow direction was kept constant. In order to prevent tracer leakage from the medium channel into the hydrogel matrix and improve microvascular network perfusability, GFP-HUVECs were seeded into the medium channels of microfluidic devices used for bead flow assay and immunostainings at day 3 or 4 according to Campisi et al. (2018). To take into account the donor variability, cells derived from three different donors for each MSCs type (BMSCs and ASCs) were tested. GFP-HUVECs cultured without any supporting cells (hereafter referred to as “EC alone”) were prepared and cultured at a final cell density of 5 million cells/ml in the same manner and served as a control for evaluating microvasculature parameters and vasculature perfusability.

### Immunofluorescent Staining

The immunostaining protocol was adapted from Honkamäki et al. (2021) and performed on 2–4 technical replicates for each cell donor. After 6 days of culture microvascular networks were washed with DPBS and fixed with 4% paraformaldehyde (Sigma-Aldrich) in DPBS for 30 min at RT following by three washes with DPBS. The samples were then permeabilized with 0.1% Triton X-100 (Sigma-Aldrich, St. Louis, MI, United States) for 10 min and non-specific binding was blocked with blocking buffer [10% normal donkey serum (NDS; Millipore), 1% bovine serum albumin (BSA; Sigma-Aldrich, St. Louis, MI, United States), 0.1% Triton X-100 in DPBS] for 2 h at RT. We used primary antibodies against vascular endothelial cadherin (VE-cadherin; BD Pharmingen, 555,661; 1:200) to identify the presence of endothelial adherens junctions, collagen type IV (Sigma-Aldrich, C1926-.2ML; 1:150) as a marker of the basement membrane, and alpha-smooth muscle actin (*α*-SMA; Abcam, ab7817; 1:300) and PDGFR-beta (PDGFR-*β*; Abcam, ab32570; 1:100) as perivascular cell markers. Primary antibodies were diluted in PBS with 1% NDS, 1% BSA, 0.1% Triton X-100 and applied for 3 days at 4°C. The samples were then washed for at least 6 times with 1% BSA in DPBS over a period of 2 days in total. Alexa Fluor 568 conjugated secondary antibodies (Thermo Fisher, A11031 and A10042) diluted 1:400 in DPBS with 1% BSA were applied overnight at 4°C. The samples were washed 5 times over a period of 2 days, stained with 0.67 µg/ml DAPI for 2 h at RT followed by three washes in DPBS. The chips were imaged using a Zeiss LSM 800 laser scanning confocal microscope with high-sensitivity two-channel spectral PMT detector and LSM 780 laser scanning confocal microscope with a Quasar spectral GaAsP detector (all from Carl Zeiss) and processed with Fiji software (Schindelin et al., 2012) and Adobe Photoshop 2020. Negative controls for immunocytochemical stainings are shown in Supplementary Figure S7.

### Microvascular Network Perfusion

To assess perfusability of the microvascular networks, 2 µm yellow-green fluorescent microbeads (FluoSpheres Carboxylate-Modified Microspheres, 2% solids, Thermo Fisher) were introduced as a fluorescent tracer into microfluidic devices containing living cells at day 7 of culture and time-sequential images were captured. Briefly, medium was aspirated from all reservoirs, 25 µl of sterile water was added into the reservoirs of one channel and 50 µl was added into the reservoirs of the other channel followed by 50 µl of fluorescent microbeads diluted 1:2,000 in sterile water. Sterile water was used instead of DPBS to avoid the formation of microbead aggregates. The generated hydrostatic pressure between the two medium channels resulted in luminal flow through the microvascular network. The time-sequential images were acquired every 10 s during 3 min using EVOS FL cell imaging system (Thermo Fisher, Nungambakkam, TamilNadu). Animated GIF images were then generated using Fiji software.

### Quantification of Microvascular Network Parameters

Microvascular networks morphology was analyzed using Fiji software. The GFP signal from the endothelial cells was used to quantify vascular area percentage, vascular network length, and average vessel diameter. The samples were fixed at day 6 and confocal z-stacked images were captured with 5 µm step (45 slices) using a Zeiss LSM 800 confocal microscope. Three regions of interest (ROIs; 798,63*798,63 µm^2^) per chip were selected from the co-culture area excluding the hydrogel-restricting microposts and solely including microvascular networks. Quantitative image analysis was not performed blinded. Images were processed as described previously (Jeon et al., 2014; Campisi et al., 2018; Haase et al., 2019) with minor modifications. Briefly, the maximum intensity projections were generated, brightness and contrast were adjusted by the “window/level” tool (35/70), noise was filtered by applying “Gaussian blur” filter (sigma 3), and images were converted to a binary format by applying the “triangle” threshold method. Artefacts were removed using the “erode” and “remove outliers” (radius 5 pixels for bright and 10 pixels for dark) functions. All values were calculated per ROI. The vasculature area percentage and the total vasculature area were computed using “measure” function. The 2D “skeletonize” function was applied to calculate the total network length. Finally, the average diameter was computed by dividing the total vasculature area by the total network length. Three chips per donor cell line and 3 ROIs per device were used for quantified analysis and statistical testing.

### Quantification of Mesenchymal Stem Cell Transition Toward Perivascular Cells

Perivascular distribution of MSCs was analyzed using Fiji software and quantified through the α-SMA signal in terms of pericyte area percentage and pericyte coverage. Immunofluorescent stained chips that had been fixed after 6 days of culture, were imaged completely with the Zeiss LSM 780 confocal microscope using Z stacks with 5 µm steps (41 slices). Maximum intensity projections were prepared and five ROIs (750*750 µm^2^) per chip were selected. Quantitative image analysis was not performed blinded. The pericyte area percentage was quantified from the *α*-SMA channel as described above for vascular parameters with minor adjustments. The pericyte coverage was quantified as the overlapping area of α-SMA and GFP signals (Erickson et al., 2020). For that, an “AND” operation of Fiji image calculator was performed on *α*-SMA and GFP channels of the same ROIs pre-processed separately. The vasculature coverage by pericytes was calculated by dividing the computed values by the total vasculature area within each ROI. Three chips per donor cell line and 5 ROIs per device were analyzed.

### Characterization of the Gravity-Driven Flow in the Microfluidic Chip

**FIGURE 1:**
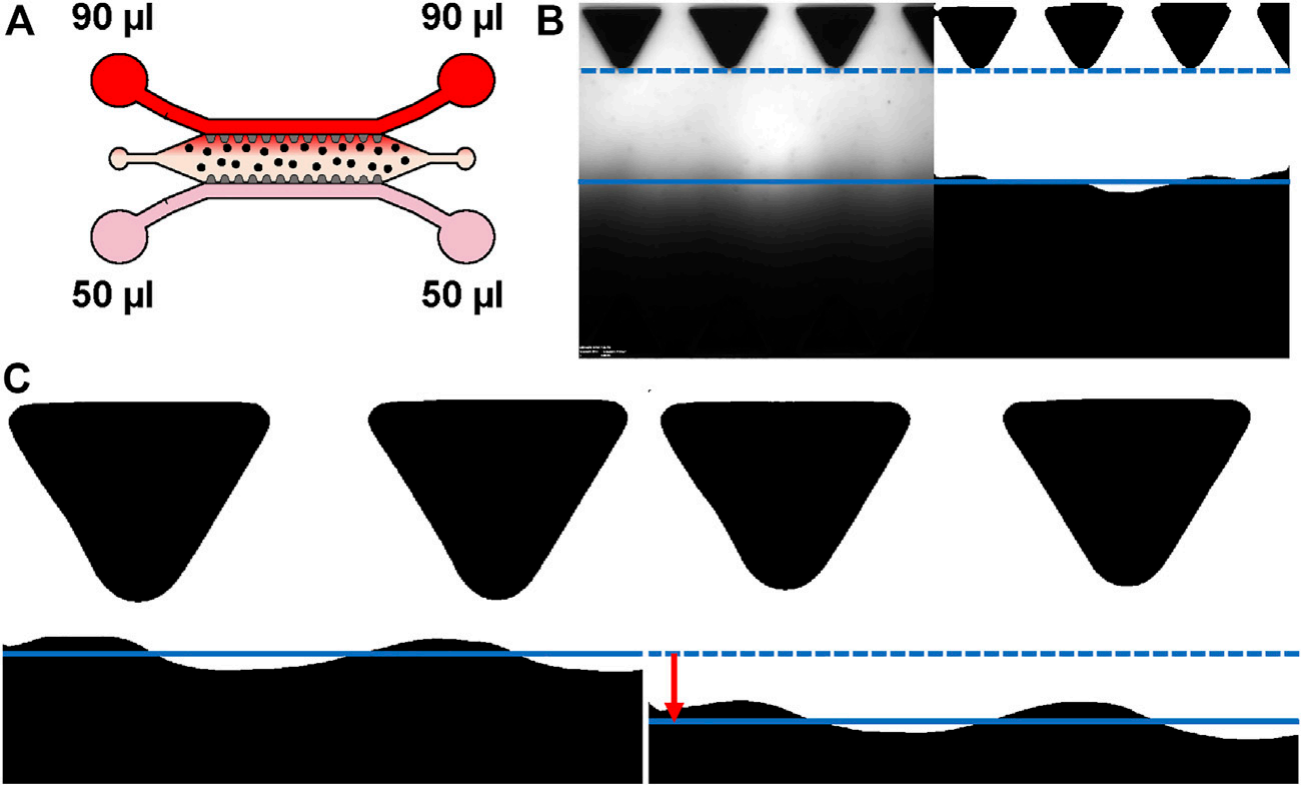
Characterizing interstitial flow within the used microfluidic chip. **(A)** Schematic representation of the experimental set-up for flow measurements. Gravity-driven flow [(90 µl + 90 µl)+(50 µl + 50 µl)] across the hydrogel area was generated by applying 50 µl of 1xDPBS to the medium reservoirs (pink) and 90 µl of 70 kDa Rhodamine B isothiocyanate–Dextran in 1xDPBS for the opposing medium reservoirs (red). Spatial change of the fluorescent wave front was imaged sequentially. Cells are depicted as dots. **(B)** Example of original and binarized image used for estimating maximal flow rate. **(C)** Example of the tracked waveforms of two consecutive images. Solid and dashed lines demonstrate the averaged waveform locations presenting the difference (red arrow) between averaged waveform locations between these two image indexes. The average change is then used to estimate current flow rate. Donor cell lines BMSC 1 and ASC 3 were used for the flow characterization co-cultures.

To characterize the gravity-driven flow generated daily, interstitial flow across the hydrogel area was measured in EC-BMSC and EC-ASC cocultures using fluorescence imaging. The flow measurements of EC-BMSCs and EC-ASCs were performed at days 0, 2, 4 and 7 using initial volumes of gravity-driven flow mimicking the flow condition. Measurements were performed at +37°C. First, medium was aspired from all reservoirs, 50 µl of 1xDPBS was added into two of the medium reservoirs (Figure 1A) followed by the addition of 90 µl of 100 µg/ml 70 kDa Rhodamine B isothiocyanate–Dextran (R9379, Sigma) in 1xDPBS to the two other reservoirs located opposite sides of the hydrogel area (Figure 1A). This generated a time-dependent hydrostatic pressure inside the chip resulting in a gravity-driven interstitial flow across the hydrogel area. To quantify the interstitial flow, sequential images of the fluorescent flow front across the hydrogel area were acquired by using a wide-field fluorescence microscope (Olympus IX 51) with sCMOS camera (Orca Flash4.0LT, Hamamatsu) using 5 seconds measurement intervals (Figure 1B). Each chip was manually placed on the microscope so that the center of the culture area was imaged. All measurements were performed as independent biological replicates N = 2 for each flow condition per coculture of one BMSC/ASC donor cell line.

Image analysis was performed using MATLAB (Version R2018b, The MathWorks, Inc., Natick, MA, United States) and scripts made in-house. First, stack images (.vsi) were read using BioFormats toolbox, greyscale images were binarized using 0.5 threshold value, and average locations of the flow front were tracked from the binarized images (Figure 1B). Based on differences in flow front locations between two consecutive indexes, known pixel size (0.615 µm), and known time interval between these two image indexes, an average flow velocity was calculated at each time point (Figure 1C, Supplementary Figure S6). An average flow rate at each time point was then calculated by multiplying the average velocities and the cross section of the hydrogel area (10,8 mm × 250 µm) (Figure 7). It should be noted that the accuracy of the developed analysis decreases within lower flow velocity, as smaller flow front movement between two consecutive image indexes is measured. This results in oscillation of the measured flow rate on day 4 (in Figure 7C), in which the flow rate was measured remarkably low (below 0.5 µl/min).

In the gravity-driven system, when assuming zero capillary forces, pressure difference *Δp(t)* between inlet and outlet reservoirs is based on time-dependent hydraulic pressure *p*_*hyd*_(*t*). As described earlier, fluorescent dextran solution was used as a tracer for determining the spatial change of the flow front preventing the possibility to record fluorescent intensity differences after the complete saturation of the hydrogel area. However, the constantly decreasing time-dependent gravity-driven flow rate thus flow duration can be estimated using following equations (Mäki et al., 2015). First, hydraulic pressure *p*_*hyd*_(*t*) is based on the liquid plug height difference *Δh*(*t*) between inlet and outlet reservoirs and can be calculated by

$$\Delta p\left(t\right)=p_{hyd}\left(t\right)=\rho g\Delta h\left(t\right)$$

where *ρ* density of liquid and *g* is gravitational acceleration.

Hydraulic resistance of the system, *R*_*hyd*_, was approximated based on measured initial flow rate from [(90 µl + 90 µl)+(50 µl + 50 µl)] volume condition and estimated initial hydraulic pressure *p*_*hyd*_ (0) using following equation. Here, it is assumed that *R*_*hyd*_ is constant during the experiment.

$$\Delta p\left(t\right)=R_{hyd}Q\left(t\right)\Rightarrow R_{hyd}=\Delta p\left(0\right)/Q\left(0\right)$$

Then, the time-dependent flow rate *Q*(t) can be estimated using equation

$$Q\left(t\right)=\frac{p_{hyd}\left(t\right)}{R_{hyd}}=\frac{\rho g\Delta h\left(t\right)}{R_{hyd}}$$

$$=\frac{\rho g\Delta h\left(t\right)}{\rho g\Delta h\left(0\right)}Q\left(0\right)=\frac{\Delta h\left(t\right)}{\Delta h\left(0\right)}Q\left(0\right)$$

In gravity-driven flow, *Δh*(*t*) is continuously decreasing as liquid flows from the inlet reservoirs to the outlet reservoirs, thus reducing the hydraulic pressure and the flow rate. With known reservoir dimensions (a cylinder shape with a diameter of 5 mm), the plug height difference between inlet and outlet reservoirs at different time points can be calculated. This equation was solved using Simulink (The MathWorks). In theory, gravity-driven flow would continue infinitely long time as the flow rate is constantly decreasing, but in practice, flow will be stopped due to small friction losses. To account this issue, a minimum flow rate threshold was set to 0.02 µl/min thus ending the simulation when the flow rate dropped below this threshold value. The simulation end-point time was then used as an estimation of total flow rate duration.

Shear stress *τ* induced by the interstitial flow velocity *v* can be estimated using equation (Yang et al., 2021)

$$\tau \approx \frac{\mu v}{\sqrt{K_{p}}}$$

in which *K*_*p*_ = 6.67 × 10^−12^ m^2^ is the permeability of the hydrogel material (Bachmann et al., 2018) and *µ* is the dynamic viscosity of the flow medium. Here, medium was assumed to have water-like properties at 37°C, thus *µ* = 0.6913 × 10^−3^ Pa s was used.

### RNA Extraction and Quantitative Real-Time PCR

At day 6 of culture, the medium was aspirated from all the media ports and chips were stored frozen at −80°C prior RNA extraction. The total RNA of EC-BMSC and EC-ASC co-cultures were extracted using TRIzol reagent (Invitrogen). Fibrin matrices containing cells were flushed out from the chips with TRIzol reagent and three chips were pooled for each BMSC/ASC donor cell line to collect higher amount of RNA. The samples were processed according to manufacturer’s instructions and RNA concentration was measured with a spectrophotometer (Nanodrop 2000; Thermo Fisher). Complementary DNA was synthesized using the High Capacity cDNA Reverse Transcription Kit (Applied Biosystems) and quantitative real time reverse transcription polymerase chain reaction (qRT-PCR) was performed with QuantStudio 12K Flex Real-Time PCR System (Applied Biosystems) using approximately 20 ng cDNA per reaction, TaqMan Fast Advanced Master Mix (Thermo Fisher) and Custom TaqMan Array Plates (Applied Biosystems) according to manufacturers’ instructions. TaqMan assay probes for genes of interest are listed in Supplementary Table S3. All measurements were performed using technical triplicates. 18S ribosomal RNA (18S), glyceraldehyde 3-phosphate dehydrogenase (*GAPDH*) and platelet and endothelial cell adhesion molecule 1 (*PECAM1*) were used as reference genes for normalizing qRT-PCR data. Gene expression analysis was performed for each EC-MSC co-cultures of three donor cell lines for both BMSCs and ASCs. Relative quantification of gene expression was performed using the 2^−ΔΔCt^ method (Livak and Schmittgen, 2001). Gene expression levels are shown relative to the average expression in EC-ASC co-cultures. Statistical analysis was performed on the mean ΔΔCt values from each donor cell line.

Heatmaps were generated from the mean ΔCt values for each donor cell line using the gplot package in R (Warnes et al., 2015). Hierarchical clustering and dendrograms were computed based on Euclidean distances.

### Statistics

All data are represented as mean ± SD. Statistical analysis was performed using GraphPad Prism 9.0.0 (GraphPad Software, www.graphpad.com). Unpaired student’s T-tests were used for significance testing between BMSCs and ASCs. *p* values were corrected to control the false discovery rate using the two-stage step-up method of Benjamini, Krieger and Yekutielio (Benjamini et al., 2006). The differences were considered statistically significant for *p* < 0.05 and represented as follows: * denotes *p* < 0.05, ** ‒ *p* < 0.01, *** ‒ *p* < 0.001, n. s ‒ non-significant.

### Data Availability Statement

The data sets generated and analyzed for the current study are available from the corresponding author on reasonable request.

## Results

### Both BMSCs and ASCs Facilitate the Formation of Mature Interconnected Microvascular Networks-On-A-Chip

**FIGURE 2:**
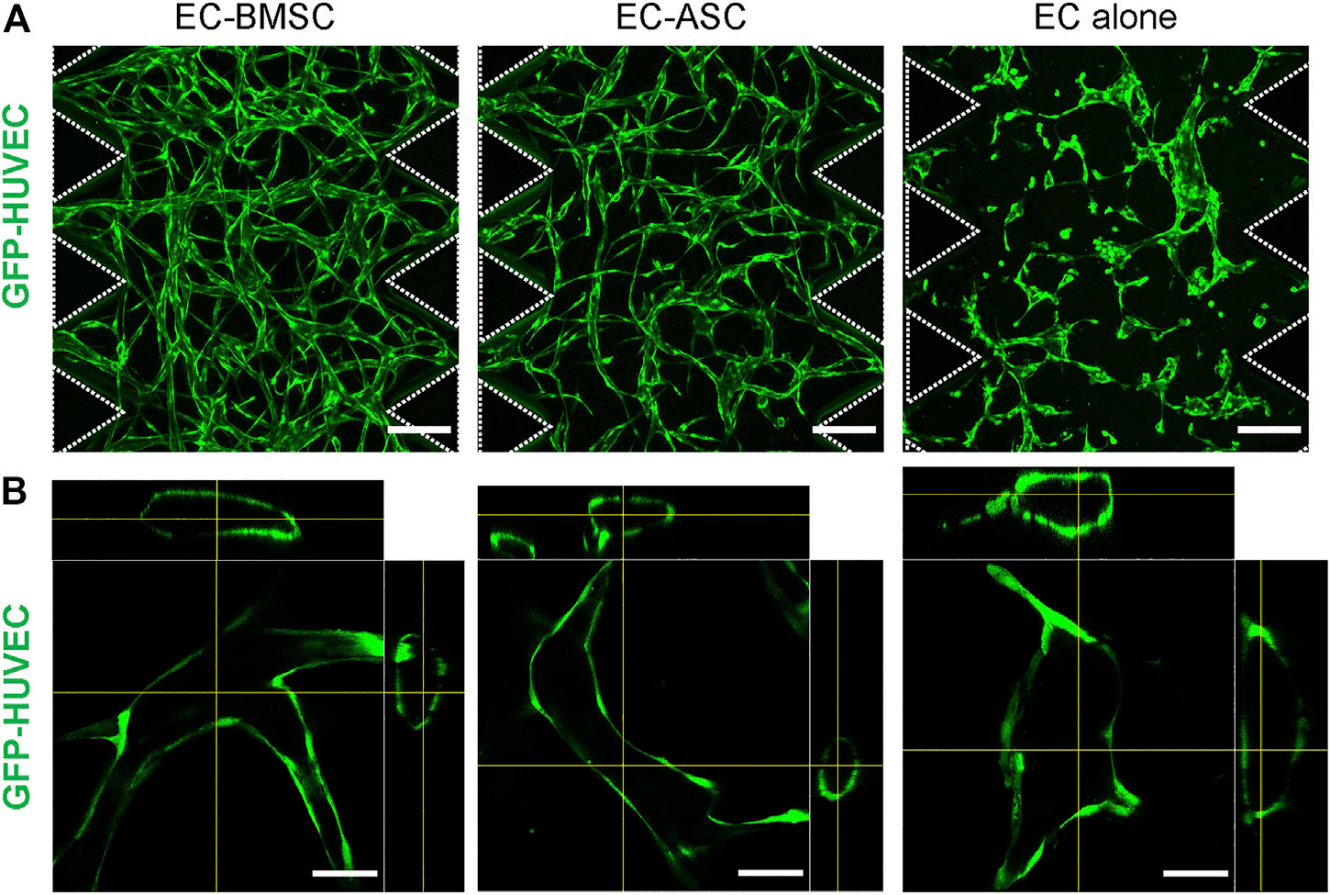
Morphology of the microvascular networks formed by EC-BMSC and EC-ASC co-cultures and ECs cultured alone. Microvascular networks were formed *via* vasculogenesis process by GFP-HUVECs cultured alone or in combination with supporting stromal cells—BMSCs or ASCs, for 6 days. **(A)** Confocal micrographs representing the overall architecture of microvascular networks established by EC-BMSC and EC-ASC co-cultures (5 million ECs/ml and 1 million MSCs/ml) and ECs alone (5 million cells/ml). Scale bars, 200 µm. Both EC-BMSC and EC-ASC co-cultures formed interconnected microvascular networks spanning the entire hydrogel over the course of 6 days. ECs cultured alone formed multicellular aggregates but failed to develop interconnected networks. **(B)** Hollow lumens could be observed in the cross-section images in all three conditions. Scale bars, 50 µm. Dash line depicts microposts that separate hydrogel from media channels. Donor cell lines BMSC 1 and ASC 2 were used for generation of data presented in the figure.

We examined two types of human primary MSCs—BMSCs and ASCs, for their ability to support microvascular network formation and maturation in fibrin matrix and act as perivascular cells when co-cultured with ECs in a microfluidic chip environment. First, we examined morphological characteristics including 3D structure, lumen-containing vessel formation, basement membrane deposition, EC-EC cell junctions of the microvascular networks formed by EC-BMSC and EC-ASC co-cultures. By day 6 both EC-BMSC and EC ASC co-cultures gradually developed into lumenized interconnected vascular networks with randomly aligned morphology spanning the entire hydrogel (Figure 2A). However, the microvascular networks formed by EC-BMSC co-cultures were more organized, interconnected, and denser compared to EC-ASC co-cultures. Daily observations of the microvascular network formation revealed minor vasculature degradation in the middle part of the chip in EC-ASC co-cultures starting from day 5 (Supplementary Figure S2).

Importantly, cross-sectional images of the vessel segments confirmed the presence of hollow lumens in both EC-BMSC and EC-ASC co-cultures as well as in EC alone (Figure 2B). In contrast to MSCs supported co-cultures, ECs cultured alone formed multicellular aggregates of different sizes which eventually developed into primitive structures containing lumens but failed to form interconnected microvascular networks. Notably, unlike in EC-BMSC and EC-ASC co-cultures, not all ECs were engaged in vasculature formation when ECs were cultured alone.

**FIGURE 3:**
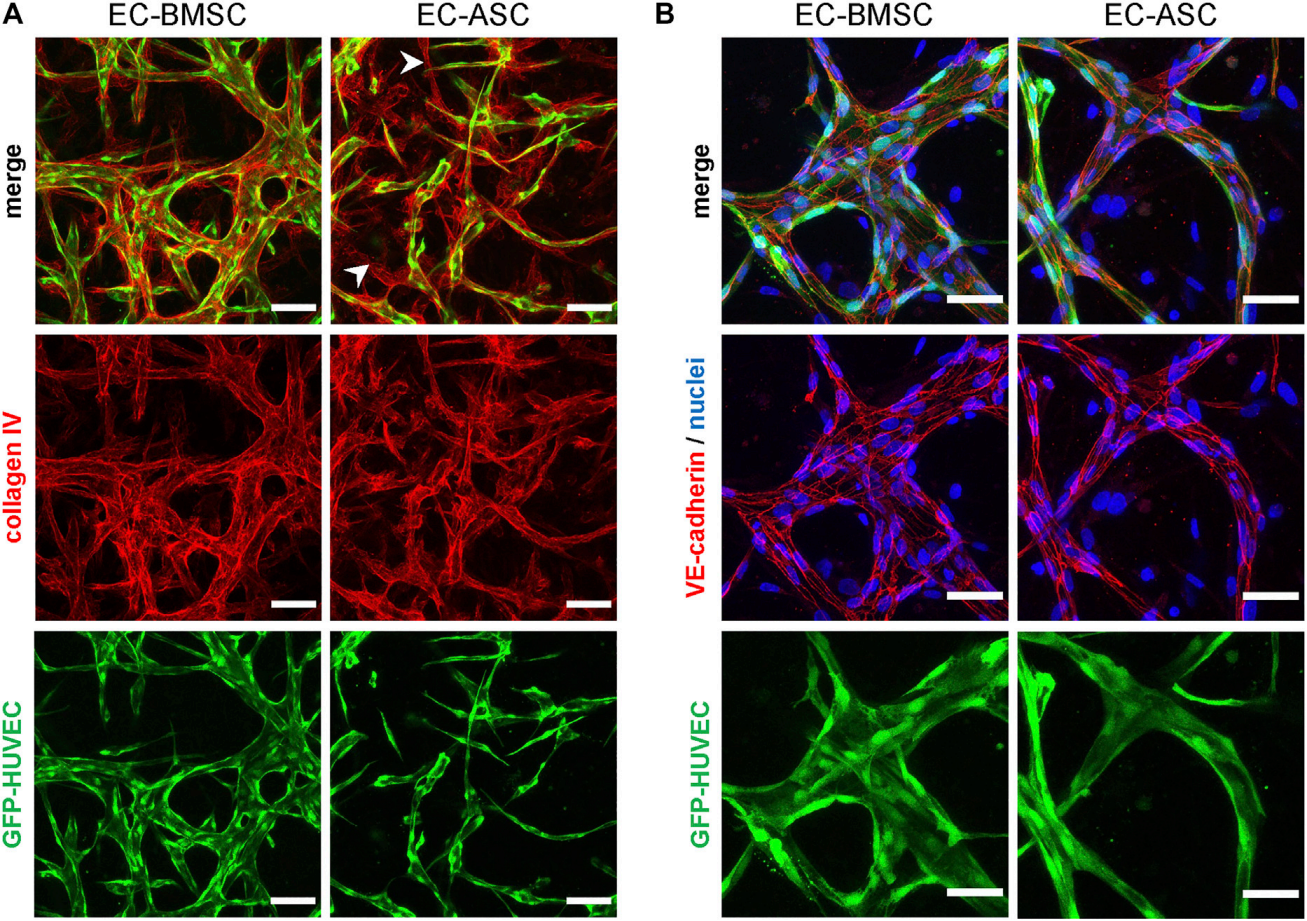
Immunocytochemistry analysis of basement membrane deposition and endothelial cell-cell junction formation in MSCs supported microvascular networks. **(A)** The EC networks (green) are surrounded by collagen IV (red), one of the major components of basement membrane, in both EC-BMSC and EC-ASC co-cultures (5 million ECs/ml and 1 million MSCs/ml). Empty basement membrane sleaves found in EC-ASC co-culture are marked by arrowheads. Scale bars, 100 µm. **(B)** Continuous and intact intercellular connections (red) are present along the border of ECs (green) in both conditions, as shown by immunostaining for adherens junction protein VE-cadherin. Nuclei (blue) are stained with DAPI. Scale bars, 50 µm. Donor cell lines BMSC 2 and ASC 1 were used for generation of data presented in **(A)**. Donor cell lines BMSC 3 and ASC 2 were used for generation of data presented in **(B)**.

We studied vascular maturation further by immunofluorescence staining for collagen type IV, a predominant vascular basement membrane component (LeBleu et al., 2007), and for VE-cadherin, a specific marker for endothelial cell-cell adherens junctions (Giannotta et al., 2013). Collagen type IV was deposited in the perivascular area surrounding the vessels in both EC-BMSC and EC-ASC co-cultures (Figure 3A). In EC-ASC co-cultures, however, collagen type IV staining was weaker and less prominent (Supplementary Figure S3). Moreover, here we detected regions of collagen type IV-positive basement membrane surrounding an empty lumen without ECs. VE-cadherin was strongly expressed at the intercellular contacts along the EC border in both EC-BMSC and EC-ASC co-cultures (Figure 3B) demonstrating formation of adherens junctions between ECs. No differences were observed between EC BMSC and EC-ASC co-cultures with VE-cadherin staining intensity.

### EC-BMSC Co-Cultures Form Consistently Perfusable Microvascular Networks Compared to EC-ASC Co-Cultures

Next, we examined perfusability of the microvascular networks formed by EC-BMSC and EC-ASC co-cultures, and ECs alone. The perfusability was assessed using 2 µm fluorescent microbeads after 7 days of culture. Microbeads were loaded into one of the medium channels and hydrostatic pressure drop was created between two medium channels so that the luminal flow across the microvascular network allows microbeads to travel through the established microvasculature. Only vessels that developed open lumens toward the media channels at the hydrogel-medium interface were able to carry beads through the network. To facilitate formation of microvasculature with open lumens, the ECs were also seeded into both medium channels of the microfluidic devices at day 3 or 4 and allowed to adhere to the hydrogel-medium interface and connect to the vascular network. With visual observation, we detected a great improvement in perfusability of the microvascular networks (data not shown). All three EC BMSC co-cultures resulted in formation of perfusable microvascular networks with multiple entry points upstream of the luminal flow and several exit points downstream of the luminal flow (Supplementary Video S1). In contrast, microvascular networks resulting from EC-ASC co-cultures demonstrated poor perfusability regardless of ASC line. Two ASC lines induced formation of partially perfusable microvessels (in only one chip out of 3 tested) with a couple entry points and only one exit point for microbeads (Supplementary Video S2). As expected, EC alone did not form perfusable microvascular networks as shown by bead flow assay (Supplementary Video S3).

### Quantification of Microvascular Networks Parameters Reveals Significant Differences in EC-BMSC Co-Cultures Compared to EC-ASC Co-Cultures

**FIGURE 4:**
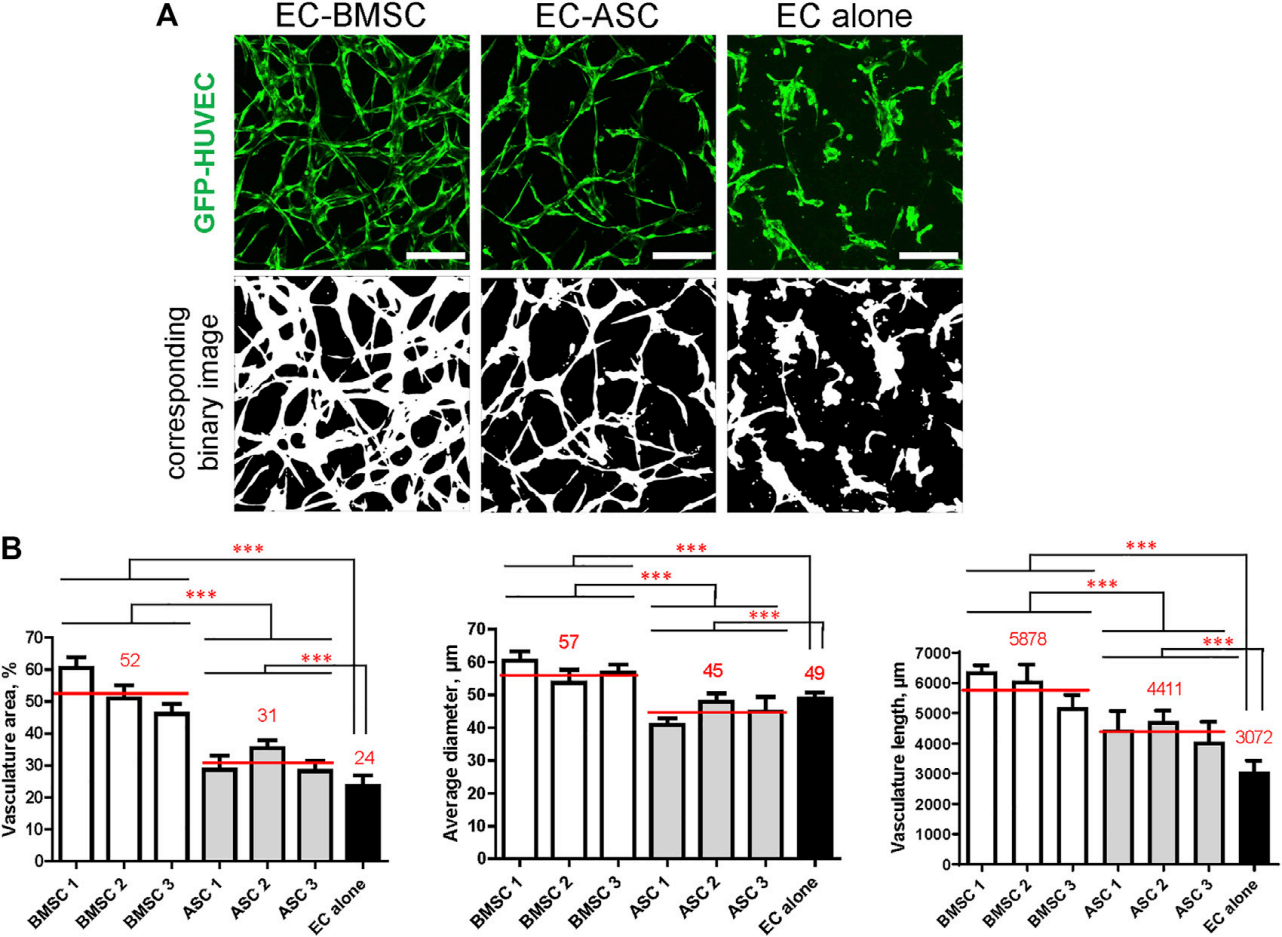
MSCs of different origin induce microvascular network formation in ECs with significantly different vasculature area, vessel diameter, and vasculature length. The presence of MSCs greatly improves microvasculature parameters compared to ECs cultured alone. **(A)** Representative images of microvascular networks derived from EC-BMSC and EC-ASC co-cultures and EC alone at day 6 and corresponding binary images. Scale bars, 200 µm. **(B)** Morphological parameters (vasculature area, average vessel diameter, and vasculature length) of MSCs supported microvascular networks are shown individually for BMSCs and ASCs derived from different donors. Data are presented as means of 9 ROIs (3 devices, 3 ROIs per device). Comparison between BMSCs and ASCs as well as comparison of BMSCs and ASCs to EC alone was performed on mean values from EC-BMSC and EC-ASC co-cultures (3 donors, 3 devices per donor, 3 ROIs per device). ***—*p* < 0.001 with Unpaired student’s *t*-test. *p* values were corrected to control the false discovery rate. Donor cell lines BMSC 2 and ASC 1 were used for generation of data presented in **(A)**.

Quantifying microvascular network parameters revealed a significantly larger vasculature area, wider vessels, and greater total vasculature length in EC-BMSC co-cultures compared to EC-ASC co-cultures (Figure 4). Microvessels occupied 52% (46–60%) of area in EC-BMSC co-cultures and 31% (28–35%) of area in EC-ASC co-cultures, while those occupied 24% of area in EC alone. As expected, MSCs supported microvascular networks covered larger area than ECs alone. EC-BMSC co-cultures formed microvascular networks with an average vessel diameter of 57 µm (54–60 µm) and a total vasculature length of 5,877 µm (5,288–6,373 µm). The average vessel diameter in EC-ASC co-cultures was 45 µm (41–48 µm) and the total vessel length 4,411 µm (4,048–4,731 µm). The average vessel diameter in EC monocultures was 49 µm and the total vessel length 3,072 µm.

### BMSCs Have More Potential to Function as Perivascular Cells and Interact With the Vascular Network than ASCs

**FIGURE 5:**
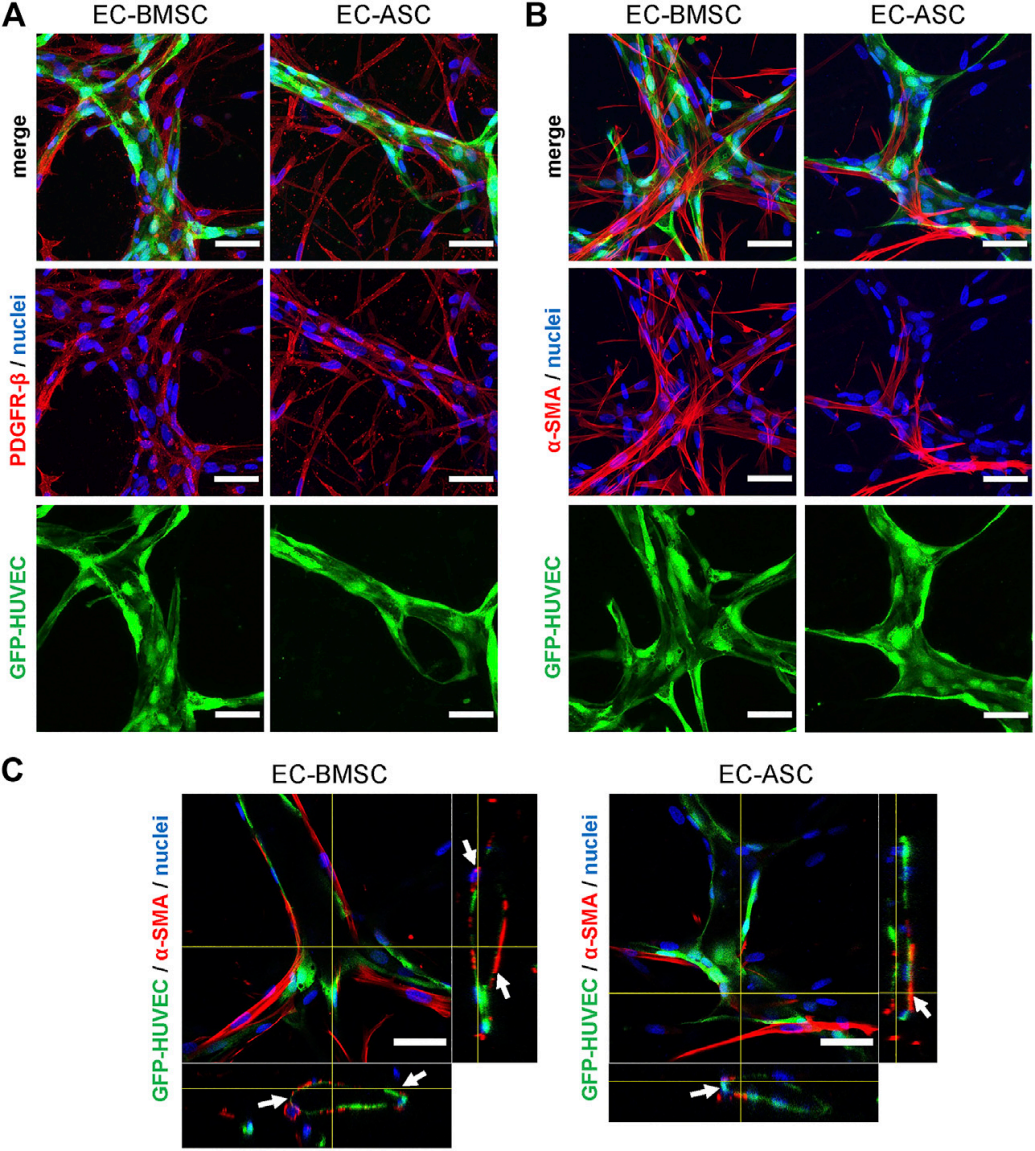
Mesenchymal stem cell transition towards perivascular cells. **(A,B)** BMSCs as well as ASCs have pericytic characteristics marked by positive expression of PDGFR-*β* **(A)** and α-SMA **(B)**. The immunostaining revealed that majority of MSCs are stained for PDGFR-*β* in both co-cultures. BMSCs are more organized and localize closely to the microvessels whereas ASCs have random appearance scattered throughout the hydrogel. In contrast to PDGFR-*β* staining, considerably less MSCs were stained for a-SMA in EC-ASC co-culture compared to EC-BMSC co-culture. Scale bars, 50 µm. **(C)** *α*-SMA positive MSCs (red) localize in close proximity and wrap around the vascular structures (green) in both EC-BMSC and EC-ASC co-cultures. White arrowheads indicate interaction of pericytes (red) with ECs (green). Scale bars, 50 µm. Nuclei (blue) are stained with DAPI. Donor cell lines BMSC 3 and ASC 1 were used for generation of data presented in **(A)**. Donor cell lines BMSC 1 and ASC 2 were used for generation of data presented in **(B, C)**.

To investigate the spatial distribution of BMSCs and ASCs as perivascular cells, the EC-BMSC and EC-ASC co-cultures were stained for known perivascular cell markers (Armulik et al., 2011; Holm et al., 2018; Sweeney and Foldes, 2018) PDGFR-*β* and α-SMA after 6 days of culture. Immunofluorescence staining showed that both BMSCs and ASCs express PDGRF-*β* and *α*-SMA when co-cultured with ECs (Figures 5A,B). Nearly all BMSCs and ASCs stained positively for perivascular cell marker PDGFR-*β*. Interestingly, BMSCs were found in close proximity to the vessel structures while ASCs were more frequently localized in areas distant from the vessels. On the contrary, immunostaining for *α*-SMA differed notably between EC-BMSC and EC-ASC co-cultures. A large fraction of BMSCs stained positively for *α*-SMA, whereas only few ASCs expressed *α*-SMA. The *α* -SMA positive MSCs had elongated morphology similar to *in vivo* and were wrapped around the microvessels or localized closely to the ECs in both EC-BMSC and EC-ASC co-cultures (Figures 5B,C).

**FIGURE 6:**
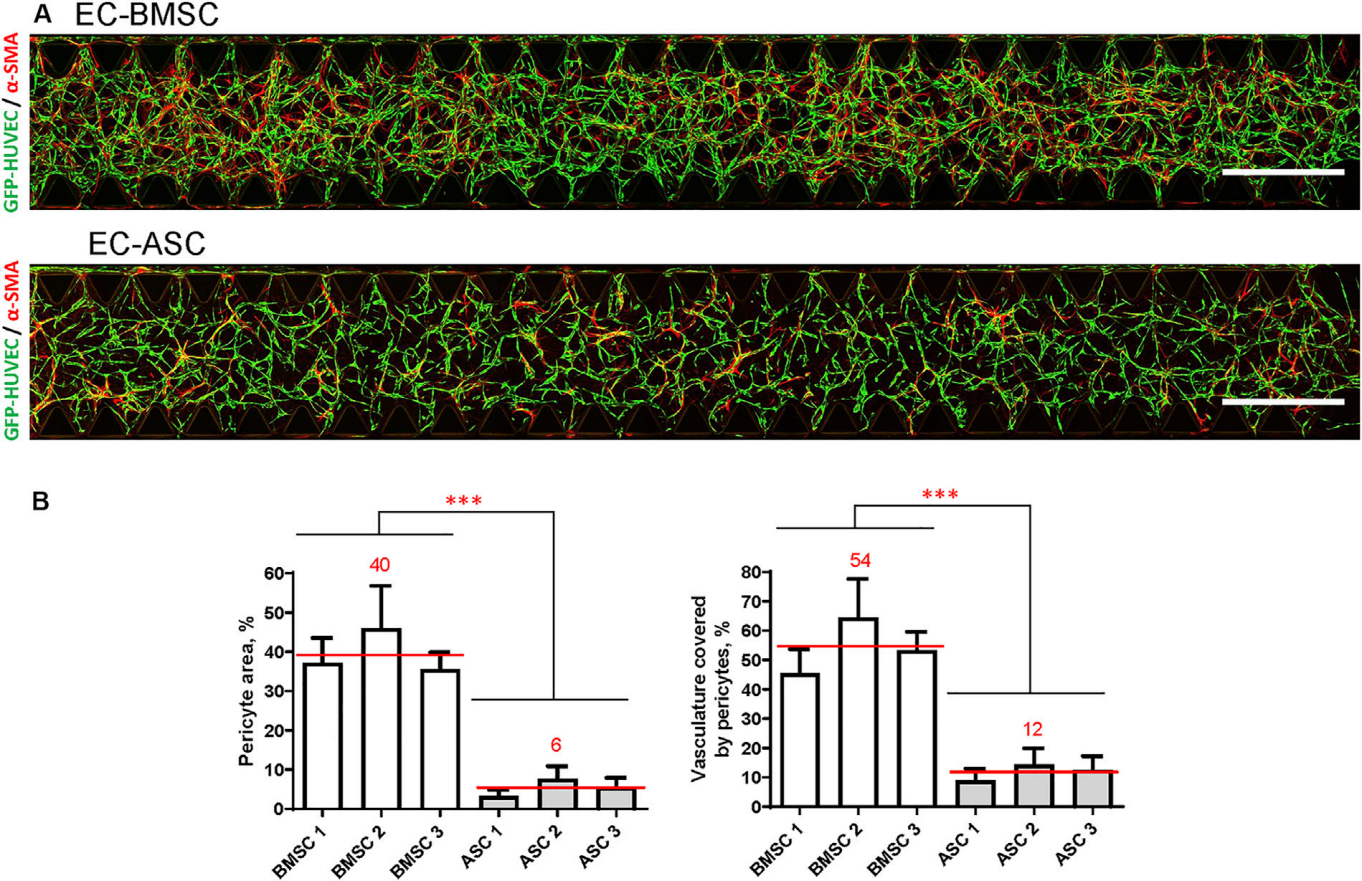
BMSCs have greater pericytic capacity when co-cultured with ECs in a microfluidic chip compared to ASCs based on significantly higher pericyte area and pericytes coverage. **(A)** Whole chip scans showing alignment of perivascular MSCs (red, marked by *α*-SMA staining) with vasculature (green) in EC-BMSC and EC-ASC co-cultures at day 6. Images are maximum intensity projections of confocal stacks 200 µm height. Scale bars, 1 mm. **(B)** Quantifications of pericyte area and pericyte coverage (vasculature covered by pericytes) are shown individually for BMSCs and ASCs derived from different donors. Data are presented as means of 15 ROIs (3 devices, five ROIs per device); error bars, SD; *** denotes *p* < 0.001 with Unpaired student’s *t*-test. Comparison between BMSCs and ASCs was performed on mean values from EC-BMSC and EC-ASC co-cultures (3 donors, 3 devices per donor, 5 ROIs per device). Donor cell lines BMSC 1 and ASC 2 were used for generation of data presented in **(A)**.

We further quantified MSCs transition toward perivascular cells using the *α*-SMA signal in terms of pericyte area percentage and pericyte coverage (Figures 6A,B). Quantification revealed that *α*-SMA positive BMSCs occupied an area (40%; 36–46%) that was more than 7-fold larger than the area occupied by *α*-SMA positive ASCs (6%; 3–8%) indicating stronger potential of BMSCs to function as perivascular cells compared to ASCs. We found that in EC-BMSC co-cultures, 54% (45–64%) of the vascular network was covered by *α*-SMA positive perivascular cells whereas in EC-ASC co-cultures – 12% (9–14%), suggesting that BMSCs have more potential to interact with and stabilize microvascular networks.

### Flow Characterization Demonstrated the Presence of the Gravity-Driven Flow Across the Hydrogel Area

**FIGURE 7:**
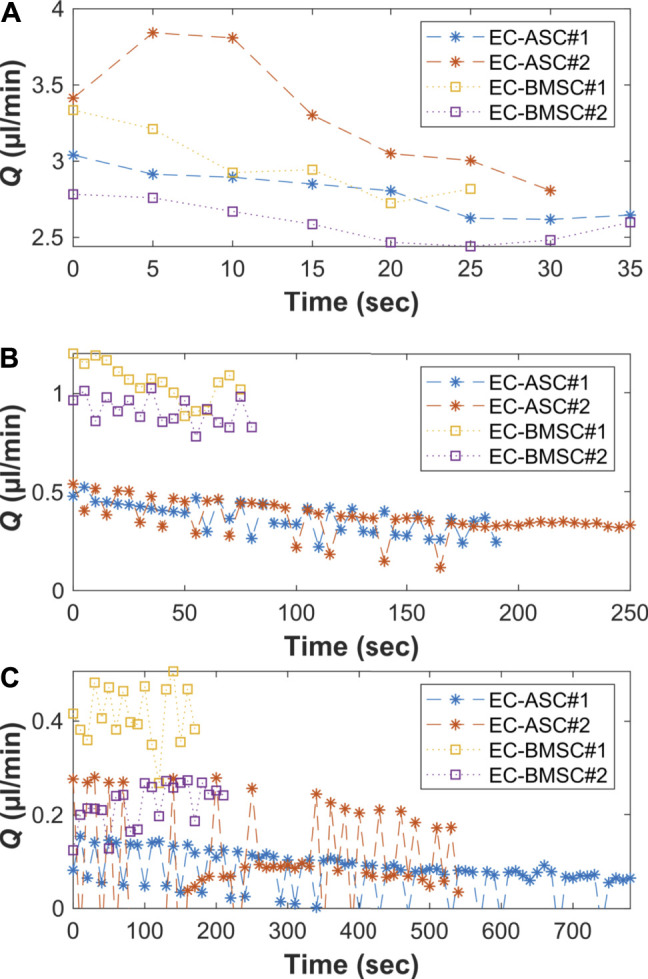
Measured average flow rates (Q) [(90 µl + 90 µl)+(50 µl + 50 µl)] on two independent biological replicates (N = 2) on **(A)** day 0, **(B)** day 2, and **(C)** day 4 for both EC-ASC (*) and EC-BMSC (□) co-cultures. **(C)** At day 4, there is minimal flow front movement between two consecutive image indexes due to decreased flow rate. This results in reduced accuracy of the used analysis method that is seen as oscillation of the measured flow rate values. Donor cell lines BMSC 1 and ASC 3 were used for the flow characterization co-cultures.

**FIGURE 8:**
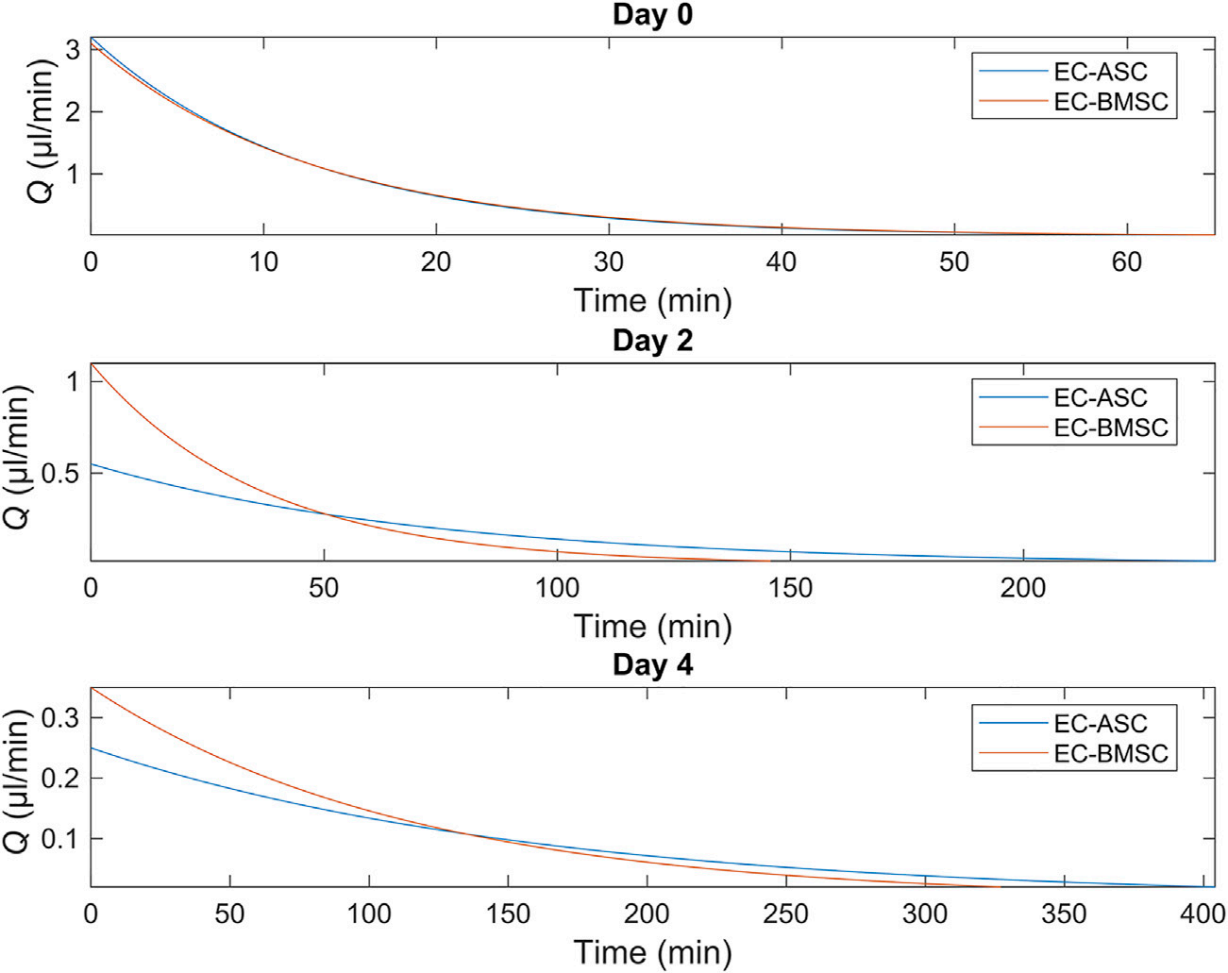
Simulated flow profiles for EC-ASC (blue) and EC-BMSC (red) co-cultures at days 0, 2 and 4 show a general decrease in flow rates and increase in total flow duration from day 0–4. Donor cell lines BMSC 1 and ASC 3 were used for the flow characterization co-cultures.

To demonstrate the presence of gravity-driven interstitial flow, we estimated the average flow rates by measuring spatial change of the wave front and computed the flow duration within the microfluidic chip using the gravity-driven model, both presented in Section *Characterization of the Gravity-Driven Flow in the Microfluidic Chip* (Figure 7). We verified the presence of gravity-driven flow across the hydrogel area within the microfluidic chip. At day 0, approximated initial flow rates *Q* (0) were 3.1 µl/min in EC-BMSC culture and 3.2 µl/min in EC-ASC culture (Figure 7A). Based on this, the estimated flow durations, where *Q*(*t*) > 0.02 µl/min, were approximately 1 h in both co-cultures (Figure 8). At day 2, measured *Q* (0) were approximately 1,1 µl/min and 0.55 µl/min (Figure 7B), prolonging the estimated flow durations from 1 h to two and 4 h for EC- BMSC and EC-ASC, respectively (Figure 8). At day 4, *Q* (0) were 0,35 µl/min and 0,25 µl/min (Figure 7C), resulting in >5 h and >6 h of estimated total flow duration for EC- BMSC and EC-ASC cultures, respectively (Figure 8). Flow characterization measurement for day 7 demonstrated unequal fluorescence wave front as we observed luminal flow *via* the formed vascular network (Supplementary Figure S6). The measured maximum interstitial flow velocity was *v* ≈ 0.024 mm/s, resulting in maximal shear stress *τ* ∼ 0.0065 Pa, which falls within the physiological shear stress range (Yang et al., 2021).

### Gene Expression Analysis Reveals Differences in Pericyte-Specific and Maturation Related Gene Expression Between EC-BMSC and EC-ASC Co-Cultures

**FIGURE 9:**
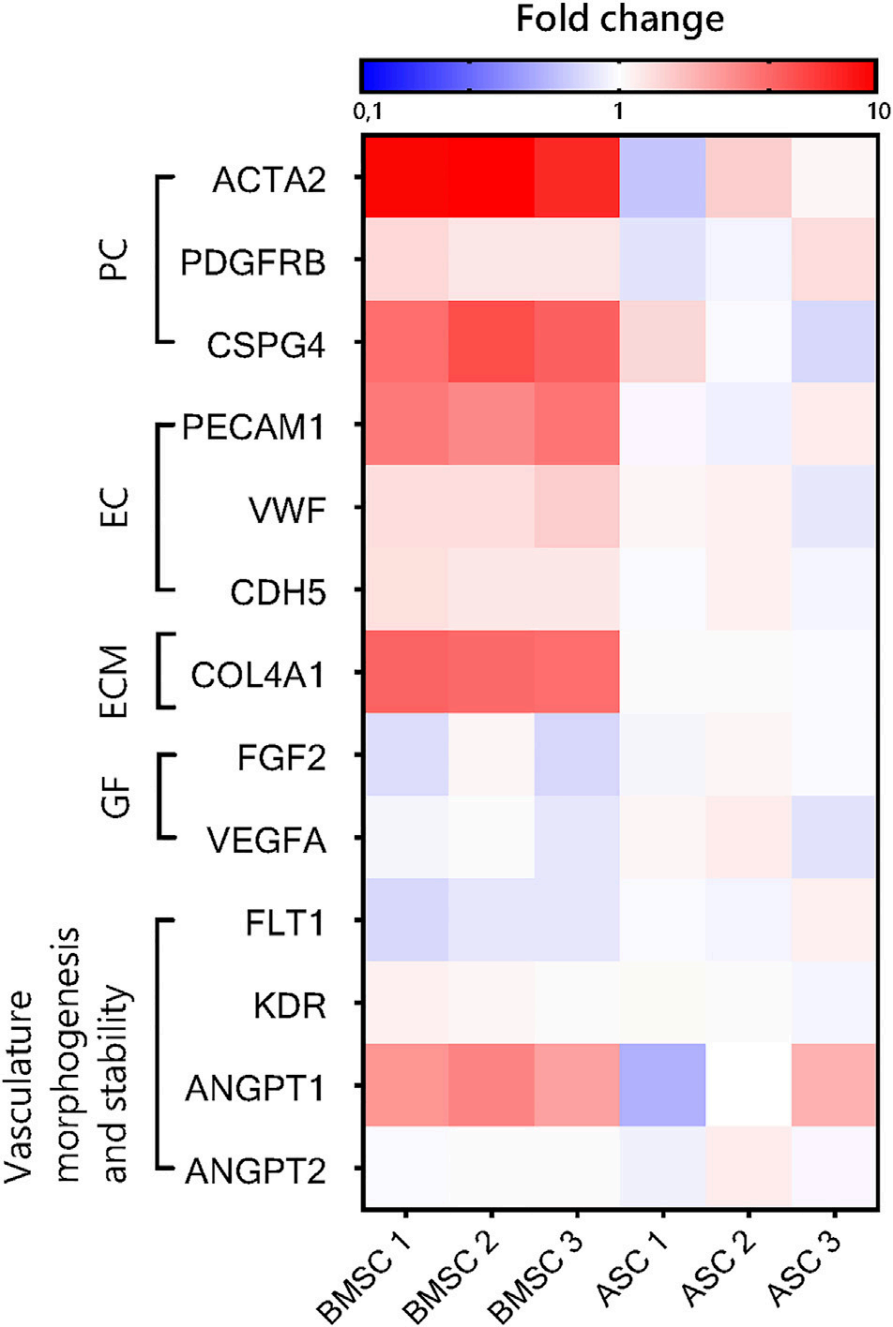
Quantitative RT-PCR analysis of gene expression in ASC- and BMSC-supported microvascular networks. Heatmap shows relative mRNA expression of pericyte-specific (PC) and endothelial-specific (EC) genes, ECM protein (ECM), angiogenic growth factors (GF), and other genes involved in vasculature morphogenesis and stability in EC-ASC and EC-BMSC co-cultures after 6 days of culture. Gene expression fold change of EC-BMSC and EC-ASC cocultures was computed relative to the average expression in EC-ASC co-cultures. The reference genes were 18S and GAPDH (for PECAM1, VEGFA, PDGFRB, CSPG2, ACTA2, ANGPT1, FGF2) and PECAM1 (for VWF, CDH5, KDR, FLT1, ANGPT2).

To investigate differences in gene expression between EC-BMSC and EC-ASC co-cultures, we performed qRT-PCR analysis for both co-cultures considering pericyte-specific (*ACTA2*, *PDFGRB*, *CSPG4*) and endothelial-specific (*PECAM1*, *CDH5*, *VWF*) genes, basement membrane protein (*COL4A1*), angiogenic growth factors (*VEGFA*, *FGF2*), and other genes involved in vasculature morphogenesis and stability (*KDR*, *FLT1*, *ANGPT1*, *ANGPT2*) (Figure 9, Supplementary Figure S4). The mRNA expression of *ACTA2*, *PDFGRB*, *CSPG4*, *PECAM1*, *COL4A1*, *VEGFA*, *FGF2*, and *ANGPT1* in both co-cultures were measured relative to 18S and *GAPDH*. To assess changes in the number of ECs between EC-BMSC and EC-ASC co-cultures, we measured the mRNA expression of *PECAM1* relative to 18S and *GAPDH*. Expression of endothelial cell specific-genes *VWF*, *CDH5*, *KDR*, *FLT1* and *ANGPT2* were measured relative to *PECAM1* (Supplementary Table S3, Supplementary Figure S4) in both co-cultures. The qRT-PCR analysis revealed a significantly higher expression of pericyte marker genes *ACTA2* and *CSPG4* (9.5-fold and 4.5-fold, respectively), also known as α-SMA and NG2, in EC-BMSC co-cultures compared to EC-ASC co-cultures (Supplementary Figure S4A). The changes in the expression of *PDGFRB* were not significant between EC-BMSC and EC-ASC co-cultures. Furthermore, expression levels of *COL4A1*, encoding for the major basement membrane protein, were 4-times higher in EC-BMSC co-cultures compared to EC-ASC co-cultures (Supplementary Figure S4C). The qRT-PCR results confirmed our observations from the immunofluorescence stainings for *α*-SMA, PDGFR-*β*, and collagen type IV.

The expression of *PECAM1*, an endothelial marker gene, was markedly higher (3.3-fold change) in EC-BMSC co-cultures (Supplementary Figure S4B). Minor differences were found in the expression of other EC-specific (*CDH5* and *VWF*) genes (Supplementary Figure S4B). The expression of *CDH5* encoding VE-cadherin was 1.3-fold higher in EC-BMSC co-cultures than in EC-ASC co-cultures. The expression of main angiogenic growth factor genes, *VEGFA* and *FGF2*, did not differ between the co-cultures (Supplementary Figure S4D). In addition, changes in gene expression of VEGFA receptors were either not significant (*KDR*) or slightly decreased in EC-BMSC co-cultures (*FLT1*, 0.8-fold change) (Supplementary Figure S4E). Considering expression of *ANGPT1* and *ANGPT2* genes, main regulators of vessel stabilization and destabilization, we found no difference in *ANGPT2* expression between EC-BMSC and EC-ASC co-cultures (Supplementary Figure S4E). Interestingly, the *ANGPT1* expression was consistently more than 2.4-times increased in EC-BMSC co-cultures. However, due to donor variation in EC-ASC co-cultures (0.5-2-fold change), the changes were not significant.

Hierarchical gene clustering showed that microvascular networks supported by BMSCs and ASCs had distinct gene expression patterns (Supplementary Figure S5). Even though expression of genes normalized by *PECAM1* (*ANGPT1*, *CDH5*, *KDR*, *FLT1* and *VWF*) was similar in EC-BMSC and EC-ASC co-cultures, BMSCs and ASCs still formed their own clusters.

## Discussion

Perivascular cells are essential for proper maturation and stabilization of the forming vascular networks (Bergers and Song, 2005; Sweeney and Foldes, 2018). Previous studies investigating 3D vasculature formation have emphasized that network phenotype and maturation strongly depend on the used perivascular cell type (Grainger and Putnam, 2011; Jeon et al., 2014; Kniebs et al., 2020; Rambøl et al., 2020; Margolis et al., 2021). While various cell types have been reported to support vasculature formation in microfluidic devices (Kim et al., 2013; Li et al., 2017; Campisi et al., 2018; Zeinali et al., 2018; Rambøl et al., 2020), differences in experimental set-up complicate the direct comparison of the results obtained with different vasculature-supporting cells. Only few studies reported systematic comparisons done for BMSCs and ASCs (Pill et al., 2018; Kniebs et al., 2020). To our knowledge, this is the first study that has compared the vasculogenic capacities of BMSCs and ASCs in a microfluidic device. Here, we used a commercially available microfluidic chip platform for co-culture of ECs with MSCs in fibrin matrices with application of interstitial flow through the hydrogel.

Our results suggest that BMSCs have stronger vasculogenic capacity and promote formation of larger and more robust vascular networks than ASCs when co-cultured with ECs in a perfusable microfluidic chip. The use of BMSCs resulted in robust and well-interconnected microvascular networks, whereas co-culture with ASCs resulted in less-interconnected vessels that occupied smaller area compared to EC-BMSC co-cultures. Though it remains unclear whether the production of basement membrane protein collagen IV in BMSCs (Sillat et al., 2012) and ASCs (Patrikoski et al., 2017) is indeed intrinsically different or whether it is due to EC-co-culture conditions, we observed more robust vasculature formed by EC-BMSC co-cultures as well as stronger basement membrane deposition compared to EC-ASC co-cultures unlike previously reported by others (Pill et al., 2018). The observed differences in vasculature phenotypes of EC-BMSC and EC-ASC co-cultures could be explained by different experimental settings used in Pill et al. (2018) study including different EC:MSC ratio, cell concentration, and, importantly, static conditions.

To explain the differences in performance between co-cultures, we investigated the expression of pericyte markers *α*-SMA and PDGFR-*β* demonstrating the distribution and alignment of MSCs with ECs (Gerhardt and Betsholtz, 2003; Sweeney and Foldes, 2018). *α*-SMA and PDGFR-*β* are known to be intrinsically expressed in both BMSCs and ASCs (Talele et al., 2015; Milan et al., 2021; Sivaraj et al., 2021). Based on the *α*-SMA and PDGFR-*β* immunostaining we could identify perivascular MSCs in our co-cultures (Parthiban et al., 2020). Our results revealed substantial differences in immunostaining for pericyte marker *α*-SMA between MSCs of different origin. While in both EC-BMSC and EC-ASC co-cultures *α*-SMA-positive pericytes aligned with ECs and were wrapped around the vessels, further quantitative analysis revealed markedly higher pericyte area and vasculature coverage by pericytes in EC-BMSC co-cultures. Interestingly, no similar pattern was observed with pericyte marker PDGFR-*β*. PDGFR-*β*-positive BMSCs localized closely to the ECs. PDGFR-*β*-positive ASCs on the other hand, were scattered around the culture area and only a small portion of ASCs aligned in close proximity to the vessels. The results suggest that BMSCs migrate more closely towards developing vascular networks, and are therefore better positioned to act as functional pericytes when compared to ASCs.

Interstitial fluid flow was applied by generating a hydrostatic pressure across the hydrogel. According to our flow characterization, we verified the presence of interstitial flow in all measured timepoints day 0, day 2, day 4 and day 7. We observed decreased maximal flow rates in day 2 and day 4 compared to day 0 in both co-cultures. The results suggest that the changes in cellular distribution and increased cell number within the fibrin matrix decrease the maximal flow rate during culture. Maximal flow rate was further decreased at day 4 which might result from ECs seeded at day 2 which form a restricting monolayer in between the media channel and the hydrogel area. We demonstrated that the existence of interstitial flow actively replenishes fresh medium for ECs and MSCs compared to static flow condition. In terms of *in vivo*-relevancy, the measured maximum interstitial flow provides physiological, interstitial shear stress of 0.0065 Pa which is in line with the previously reported range (Kim et al., 2014; Yang et al., 2021). Hence, applying interstitial flow across the hydrogel with appropriate mechanical stress enhances the physiological relevancy of our *in vitro* vascular models (Kim et al., 2016; Haase and Kamm, 2017).

To describe the general flow characteristics in our study, we verified the formation of perfusable vessels after day 4, which led into a transition of the interstitial flow into luminal flow due to conjoining perfusable vessels across the hydrogel area. According to our bead flow assay at day 7, we verified the vessel capability to carry out particles in both co-cultures. This result is in line with immunocytochemical stainings showing the structural maturation at day 6. Still, we observed a considerable difference in vasculature perfusability between the two MSC types. BMSCs supported the formation of perfusable microvascular networks in all tested devices whereas EC-ASC co-cultures resulted in partially perfusable vasculature in only 2 out of 9 tested devices. This result is important, because perfusion capability is a key property of 3D vascularized *in vitro* models (Kim et al., 2013). Overall, our results highlight that in addition to immunocytochemical analyses of vessel maturation, functional measuring e.g. network perfusability is essential to assess the quality and functionality of the engineered vascular networks.

We found substantial differences in gene expression between EC-BMSC and EC-ASC co-cultures in genes related to pericytic and angiogenic activity supporting consistently our structural analysis. Relatively high expression levels of *ACTA2* and *CSPG4* indicate strong pericytic characteristics of BMSCs in accordance with abundant *α*-SMA staining shown in this study. Consistent with our immunohistochemical analysis and previous findings (Pill et al., 2018), *PDGFRB* expression was similar between co-cultures. We measured 4-fold higher expression of *COL4A1* in EC-BMSC, verifying our observations from immunohistochemistry that BMSCs more strongly support formation of the basement membrane. However, it remains unclear whether the COL4A1 expression is intrinsically different in BMSCs and ASCs or whether it is due to co-culture with ECs. We did not find differences in the expression of main angiogenic growth factor genes *VEGFA* and *FGF2*, neither in the main vasculature destabilizing gene *ANGPT2* between co-cultures which is in accordance with previous findings (Pill et al., 2018). However, we observed a trend in consistently higher expression levels of *ANGPT1* in EC-BMSC co-culture, a gene responsible for vessel maturation and stability. The results support our observations of more mature and stable microvascular networks in EC-BMSC co-cultures.

We found that endothelial cell marker gene *PECAM1* was over three times more expressed in EC-BMSC co-cultures than in EC-ASC co-cultures at the end of the 6-days culture period. This might indicate that ECs proliferate more and survive better in EC-BMSC than EC-ASC co-cultures. However, a previous study comparing vasculature-supporting properties of BMSCs and ASCs in a bulk hydrogel did not find significant changes in the number of ECs during 21-days culture period or between the co-cultures (Pill et al., 2018).

One possible explanation for the observed differences in vascular morphogenesis between EC-BMSC and EC-ASC co-cultures could be tissue-specific characteristics of MSCs as vasculature supporting cells. Although BMSCs and ASCs share similar characteristics concerning cell morphology and surface marker expression (Bourin et al., 2013), significant biological differences have been shown for different types of MSCs, such as MSCs from bone marrow, skin dermis, and adipose tissue (Woo et al., 2016). Moreover, recent donor-matched comparison of BMSCs and ASCs revealed differences in proliferation and differentiation potential (Mohamed-Ahmed et al., 2018). It is important to remember that on macroscopic level, bone marrow and adipose tissue are remarkably different tissues. Thus, BMSCs and ASCs may differ concerning their vasculature supporting characteristics as well. Indeed, endothelial-to-pericyte ratio and vasculature coverage by pericytes vary considerably between different tissues ranging between 1:1 and 10:1 and 70 and 10% respectively (Armulik et al., 2011). For example, pericyte coverage in the bone marrow was measured to be 51 ± 20% (Zetterberg et al., 2007). Even though we measured significantly different pericyte coverage between EC-BMSC and EC-ASC co-cultures, both values fall within the range documented *in vivo*. Pericyte coverage correlates positively with endothelial barrier properties and endothelial cell turnover indicating the role of pericytes in regulating vessel permeability and stabilizing vascular networks (Armulik et al., 2011). Indeed, in our study we demonstrated higher pericyte coverage as well as higher expression of pericyte specific and maturation related genes in EC-BMSC co-cultures. Still, previous studies using other stromal cells demonstrate that altering the ratio of ECs to ASCs cell concentration, or hydrogel stiffness may be used to tailor vessel characteristics towards more robust, mature and perfusable microvasculature in EC-ASC co-cultures (Whisler et al., 2014; Tiruvannamalai Annamalai et al., 2016).

To summarize, we used a commercially available microfluidic device for generating interconnected, 3D vascular networks supported by either BMSCs or ASCs to systematically investigate the effect of chosen MSCs type on vascular network phenotype, functionality, and gene expression with commonly used HUVECs. We demonstrated that BMSCs have stronger pericytic ability and support mature vascular network formation under dynamic culture conditions. Our study demonstrates how vascular network phenotype depends on MSCs tissue source, resulting in differing gene expressions, vasculature morphology and characteristics, and ultimately, vascular network functionality. Therefore, we suggest considering these varying characteristics when developing advanced vascularized organ-on-a-chip platforms.

## Data Availability

The original contributions presented in the study are included in the article/Supplementary Materials, further inquiries can be directed to the corresponding authors.
