# Supplementary material for: Vasculogenic Potency of Bone Marrow- and Adipose Tissue-Derived Mesenchymal Stem/Stromal Cells Results in Differing Vascular Network Phenotypes in a Microfluidic Chip
Source: Front Bioeng Biotechnol. 2022 Feb 8;10:764237. doi: 10.3389/fbioe.2022.764237 (PMC8861308; doi:10.3389/fbioe.2022.764237)
Supplement: Supplementary file 3 [file DataSheet1.PDF]

Supplementary Material

Supplementary Tables

Supplementary Table 1. Donor information for the used primary mesenchymal stem/stromal cell lines BMSC 1-3 and ASC 1-3. For donor cell line ASC 1, Body Mass Index (BMI) was not available (N/A).

Donor ID	Gender	Age	BMI
BMSC 1	Female	87	28,9
BMSC 2	Female	81	23,8
BMSC 3	Male	92	19,0
ASC 1	Female	31	N/A
ASC 2	Female	47	25,0
ASC 3	Female	63	24,4

Supplementary Table 2. Surface marker expression of the studied donor BMSCs and ASCs. Individual donor cell line passage during analysis is denoted in the column ''P''. Positive > 80%; low < 10%; negative < 2%. The cells were characterized as MSCs due to positive expression of CD73, CD90, and CD105, and low or negative expression of CD14, CD19, CD45 (Dominici et al. 2006; Bourin et al. 2013). The expression of CD34 and HLA-DR was present at variable levels. The heterogeneity in surface marker expression if compared to the ISCT requirements could be explained by changes in cell culturing conditions (Patrikoski et al. 2013).

		**Surface marker expression**							
		**CD14**	**CD19**	**CD34**	**CD45**	**CD73**	**CD90**	**CD105**	**HLA-DR**
**Donor ID**	**P**	low to negative	low to negative	variable	low to negative	positive	positive	positive	variable
**BMSC 1**	3	7,4	7,1	3,1	8,8	95,4	90,2	93,6	92,2
**BMSC 2**	3	4,6	3,4	2,3	6,4	89,1	81,5	86,5	76,4
**BMSC 3**	3	5,2	2	1	3,5	95,6	86,6	97,2	96,8
**ASC 1**	1	1,2	1	78,6	4,1	97,0	98,2	99,6	1,4
**ASC 2**	1	0,7	1,2	10,8	1,5	97,2	99,2	99,6	0,9
**ASC 3**	2	7,1	7,7	11,5	8,7	99,5	99,8	100	1

Supplementary Table 3. TaqMan assay probes, function, and likely source of genes used for qRT-PCR.

Gene symbol	Gene name, alias	TaqMan probe ID	Function	Likely source
**GAPDH**	glyceraldehyde-3-phosphate dehydrogenase	Hs02786624_g1	basic enzyme	all cells
**18S**	eukaryotic 18S rRNA	Hs99999901_s1	structural RNA of eukaryotic cytoplasmic ribosomes	all cells
**PECAM1**	platelet and endothelial cell adhesion molecule 1, CD31	Hs01065282_m1	endothelial cell intercellular junctions' protein	ECs
**VEGFA**	vascular endothelial growth factor A	Hs00900055_m1	main angiogenic and vasculogenic growth factor	MSCs
**PDGFRB**	platelet derived growth factor receptor beta, CD140b	Hs01019589_m1	promote proliferation, migration and recruitment of pericytes and smooth muscle cells to endothelial cells; pericyte marker	MSCs
**CSPG4**	chondroitin sulfate proteoglycan 4, NG2	Hs00361541_g1	stimulates endothelial cells motility during microvascular morphogenesis; pericyte marker	MSCs
**ACTA2**	actin, alpha 2, smooth muscle, aorta, α-SMA	Hs00426835_g1	involved in vascular contractility and blood pressure homeostasis; marker for pericytes and smooth muscle cells	MSCs
**ANGPT1**	angiopoietin 1	Hs00919201_m1	contributes to blood vessel maturation and stability, inhibits endothelial permeability; anti-inflammatory molecule	MSCs

**ANGPT2**	angiopoietin 2	Hs00169867_m1	antagonist of angiopoietin 1; pro-inflammatory molecule	ECs
**VWF**	von Willebrand factor	Hs01109454_m1	endothelial derived thrombocyte adhesion molecule	ECs
**CDH5**	cadherin 5, vascular endothelial cadherin, CD144	Hs00901470_m1	organization of endothelial cell intercellular junctions, maintains a restrictive endothelial barrier	ECs
**KDR**	kinase insert domain receptor, VEGFR2	Hs00911700_m1	VEGFA receptor; main mediator of VEGF-induced endothelial proliferation, survival, migration, tubular morphogenesis and sprouting	ECs
**FGF2**	fibroblast growth factor 2	Hs00266645_m1	broad mitogenic and angiogenic activities, can induce angiogenesis	MSCs
**COL4A1**	collagen type IV alpha 1 chain	Hs00266237_m1	integral component of basement membranes	all cells
**FLT1**	fms related tyrosine kinase 1, VEGFR1	Hs01052961_m1	VEGFA receptor; regulation of angiogenesis and vasculogenesis: promotes PGF-mediated proliferation of endothelial cells, may function as a negative regulator of VEGFA signaling, modulates KDR signaling	ECs

Supplementary Figures

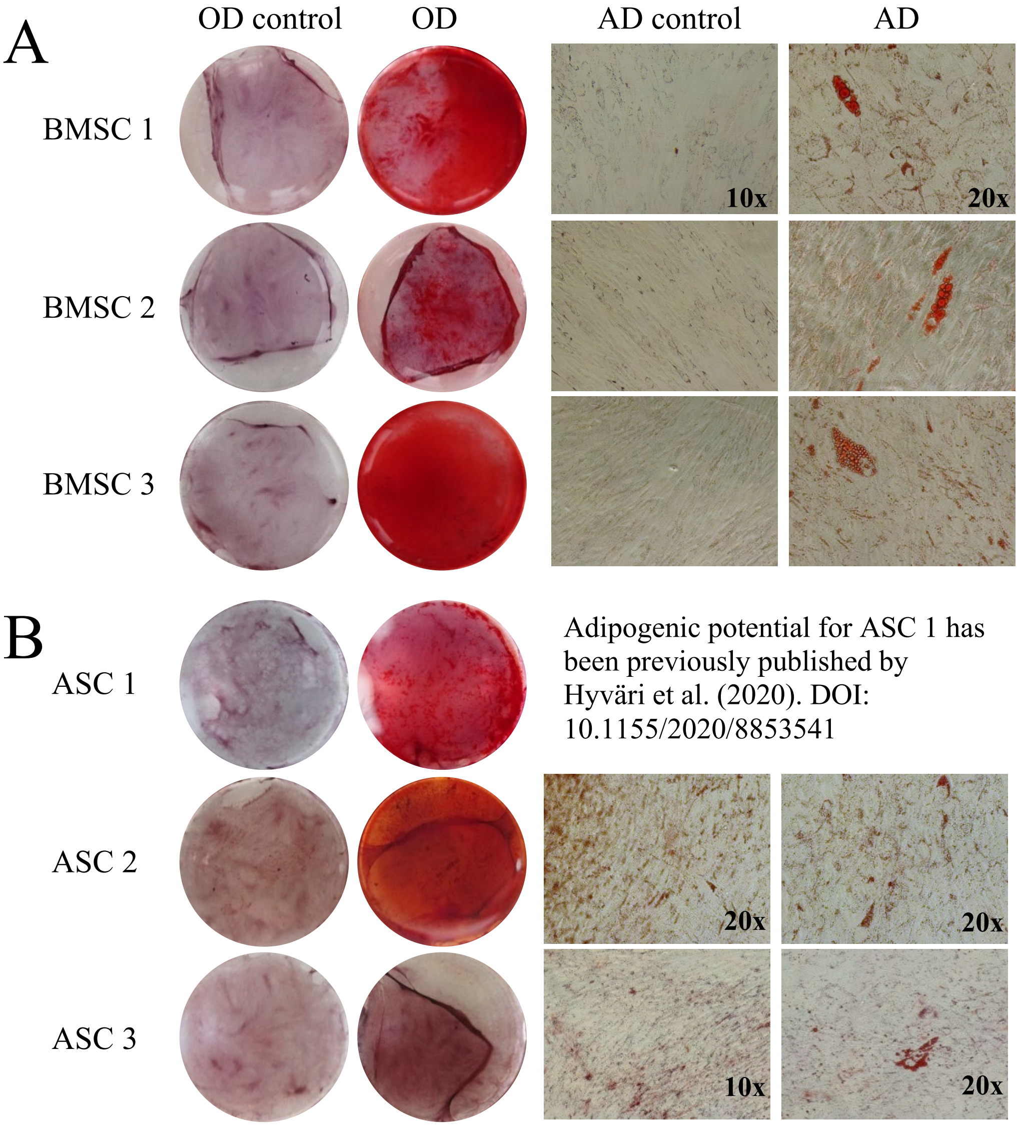

Supplementary Figure 1. Osteogenic (OD) and adipogenic (AD) differentiation of studied donor mesenchymal stem/stromal cells (MSCs). Differentiation medium compositions and differentiation cultures were performed as previously described (Hyväri et al. 2018) with minor modification as α-MEM (Gibco) was used as a basal medium instead of DMEM and 1% GlutaMAX was excluded. In OD, cells were cultured for 20-21 days in OD medium (Hyväri et al. 2018), which was changed twice a week. Differentiated MSCs were stained with Alizarin Red to demonstrate the MSCs' potency for producing mineralized calcium deposits. To examine the adipogenic potential of MSCs, cells were cultured for 7 or 14 days in AD medium (Hyväri et al. 2018), which was changed twice a week. Differentiated MSCs were stained with Oil Red O for examining the presence of intracellular lipid accumulation. MSCs cultured in control medium (α-MEM with 5% human serum (BioWest, PAA or Serana), 100 U/ml penicillin, and 100 µg/ml streptomycin) were used as a negative control. A) OD and AD potency of the studied BMSCs. B) OD and AD potency of the studied ASCs. Adipogenic potential for ASC 1 has been published previously (Hyväri et al. 2020).

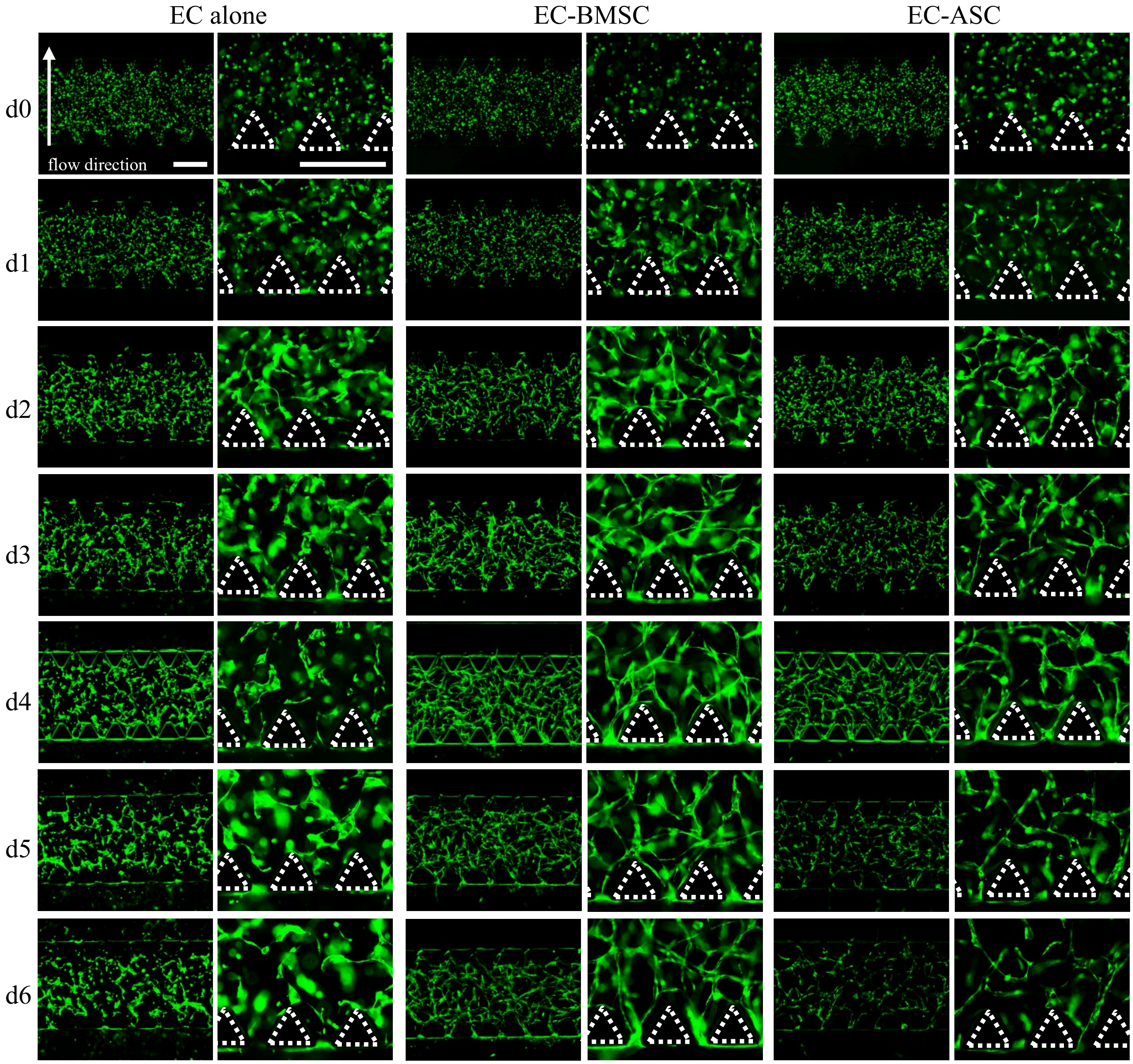

Supplementary Figure 2. Daily observations of microvascular networks development via vasculogenesis process in ECs cultured alone and in combination with supporting stromal cells – BMSCs or ASCs, in microfluidic chips. At day 0 the cells seeded into microfluidic chips are evenly distributed in all three conditions. In EC-BMSC and EC-ASC co-cultures, ECs started to form connections as early as on day 2 and gradually developed into interconnected microvascular networks spanning the entire hydrogel over the course of 6 days. Minor vascular network regression was noticed in central region of EC-ASC co-cultures starting from day 5. ECs cultured alone fail to form interconnected networks. Dash line depicts microposts that separate hydrogel from media channels. Scale bars, 500 µm. Donor cell lines BMSC 2 and ASC 2 were used for generation of data presented in the figure.

EC-BMSC EC-ASC

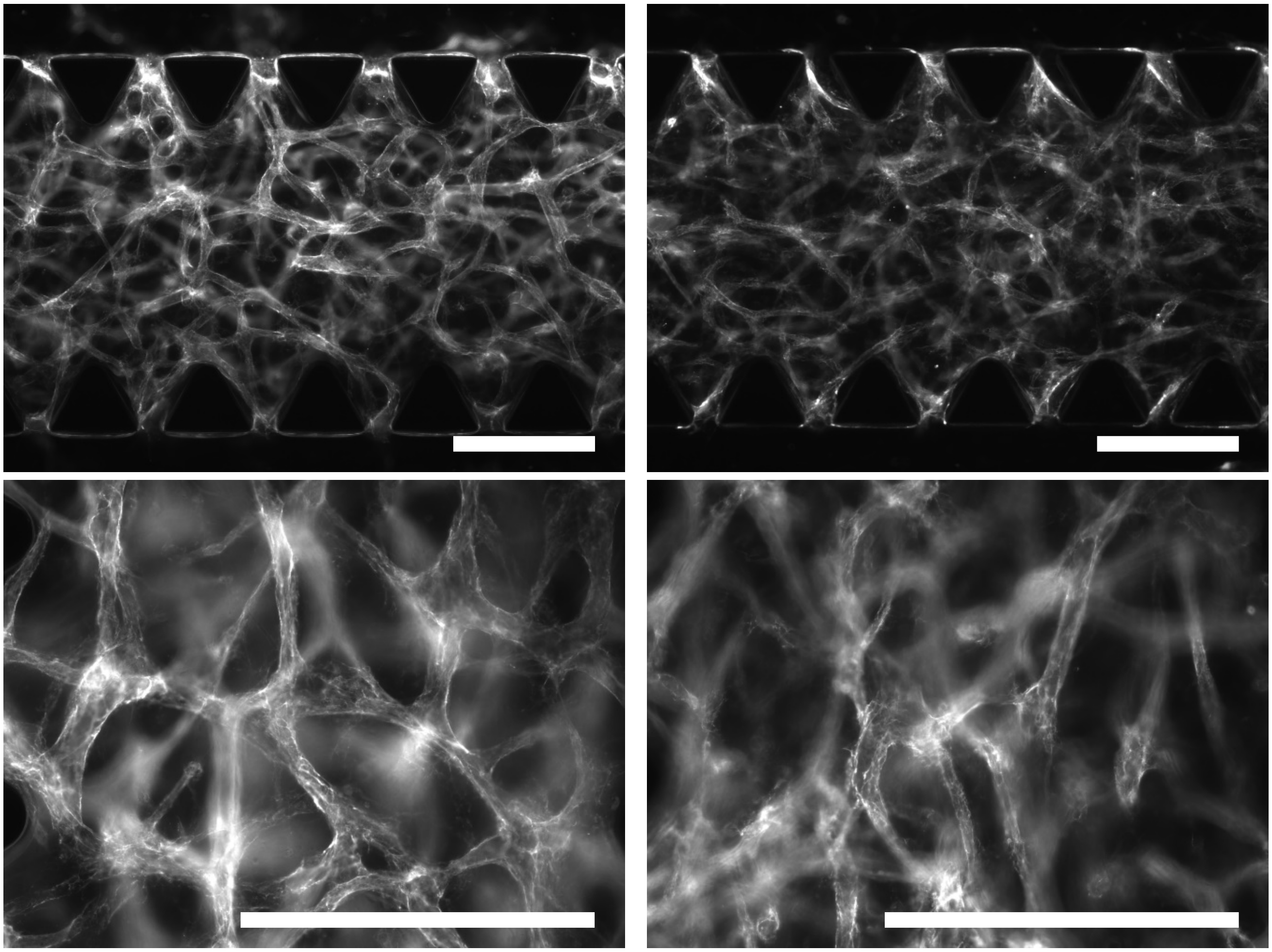

Supplementary Figure 3. Widefield fluorescence images of collagen IV expression in BMSCs and ASCs supported microvascular networks cultured for 6 days. ECM basement membrane protein collagen IV was deposited in both EC-BMSC and EC-ASC co-cultures. However, collagen IV deposition was more prominent and more organized in EC-BMSC co-culture based on greater collagen IV staining intensity, providing evidence for improved vascular function in EC-BMSC co-culture. Scale bars, 500 µm. Donor cell lines BMSC 3 and ASC 3 were used for generation of data presented in the figure.

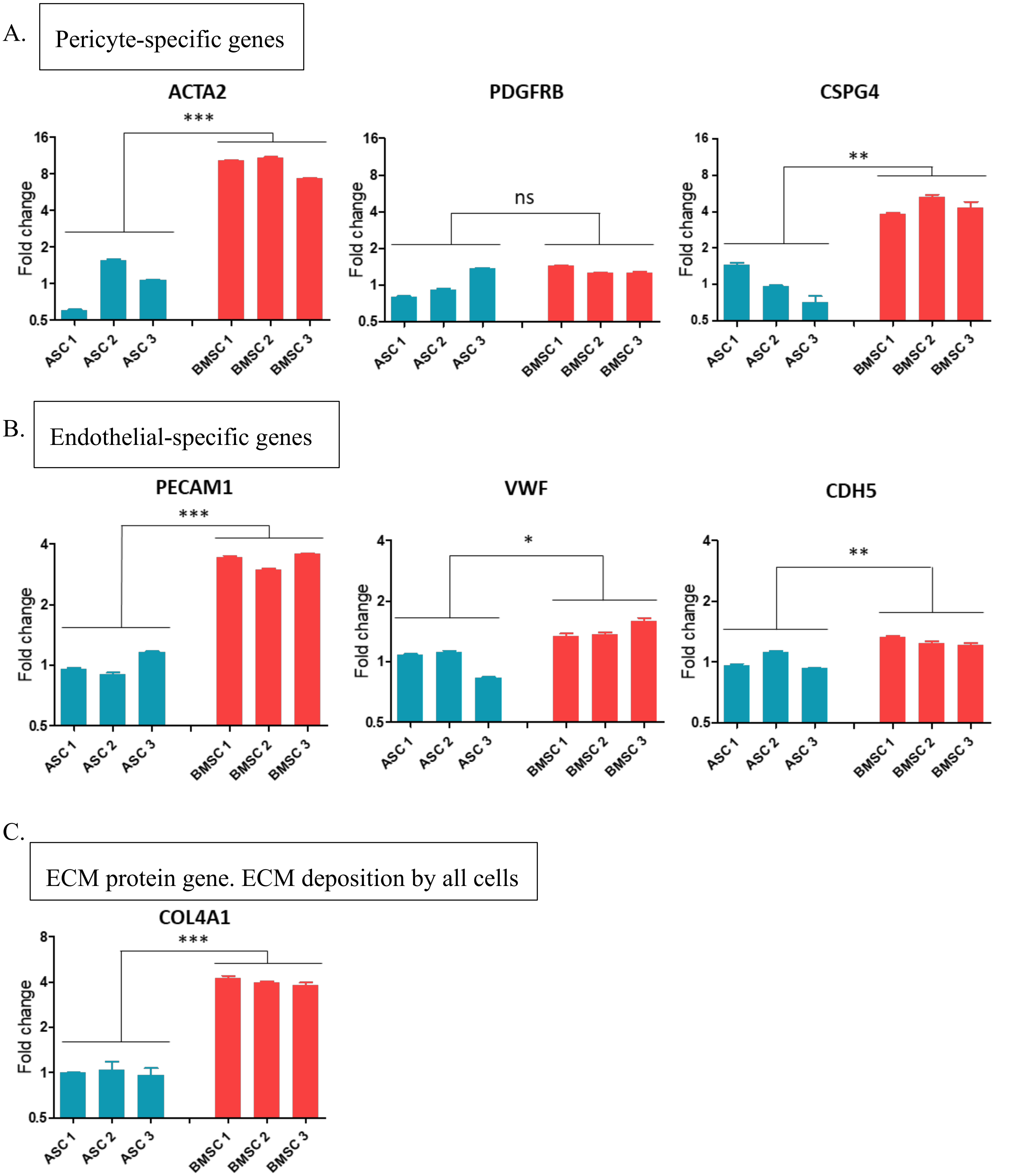
A.
Pericyte-specific genes
ACTA2
***
PDGFRB
ns
CSPG4
**
Fold change
16
8
4
2
1
0.5
ASC 1
ASC 2
ASC 3
BMSC 1
BMSC 2
BMSC 3
B.
Endothelial-specific genes
PECAM1
***
VWF
*
CDH5
**
Fold change
4
2
1
0.5
C.
ECM protein gene. ECM deposition by all cells
COL4A1
***
Fold change
8
4
2
1
0.5

D. Main angiogenic growth factors genes

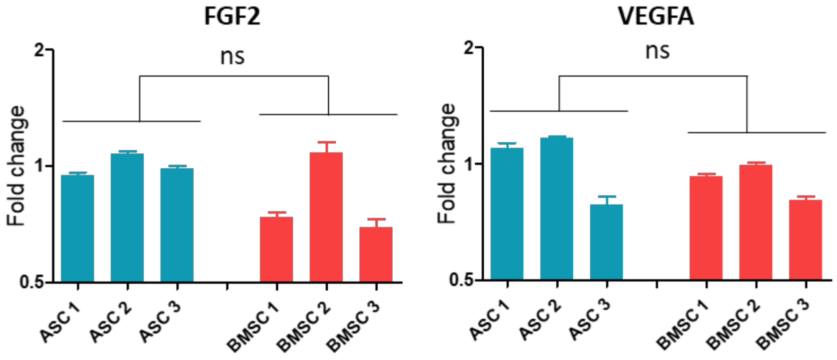

E. Genes involved in vasculature morphogenesis and stability

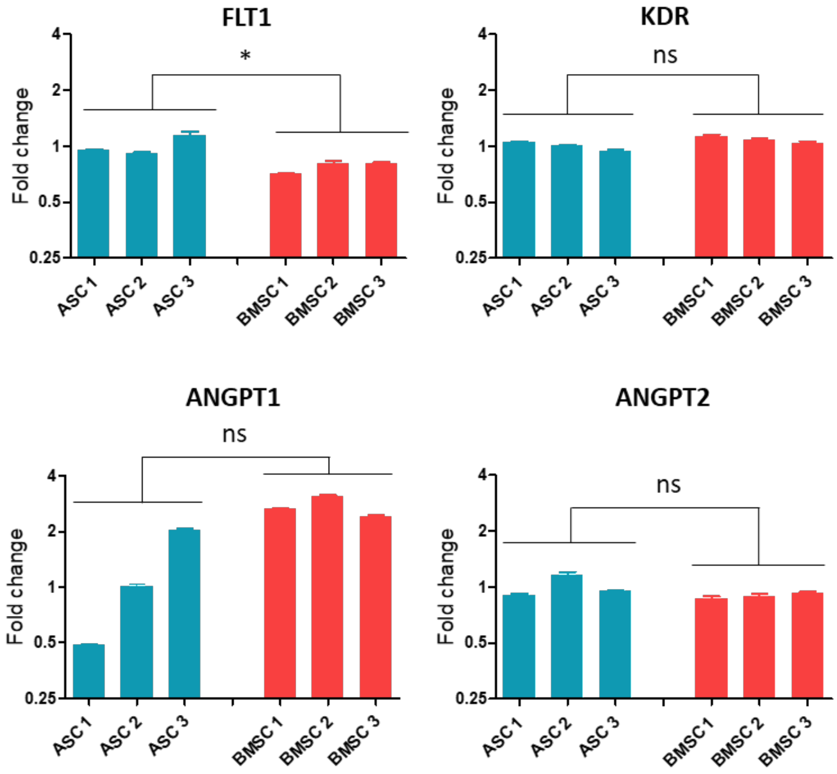

Supplementary Figure 4. Quantitative RT-PCR analysis of gene expression in ASCs and BMSCs supported microvascular networks after 6 days of culture. Comparison of mRNA expression between EC-BMSC and EC-ASC co-cultures are performed relative to the average expression in EC-ASC co-cultures. Analyzed genes are grouped by their main function based on literature data: pericyte-specific (A) and endothelial-specific (B) genes, ECM protein (C), angiogenic growth factors (D), and other genes involved in vasculature morphogenesis and stability (E). The mRNA expression was normalized to 18S and GAPDH (ACTA2, PDFGRB, CSPG4, PECAM1, COL4A1, VEGFA, FGF2, ANGPT1) or PECAM1 (VWF, CDH5, KDR, FLT1, ANGPT2). Data are presented as means of 2-3 technical replicates; error bars, SD; * denotes $p < 0.05$, ** – $p < 0.01$, *** – $p < 0.001$, n.s. – non-significant with Unpaired student's T-test. P values were corrected to control the false discovery rate.(Benjamini, Krieger, and Yekutieli 2006)

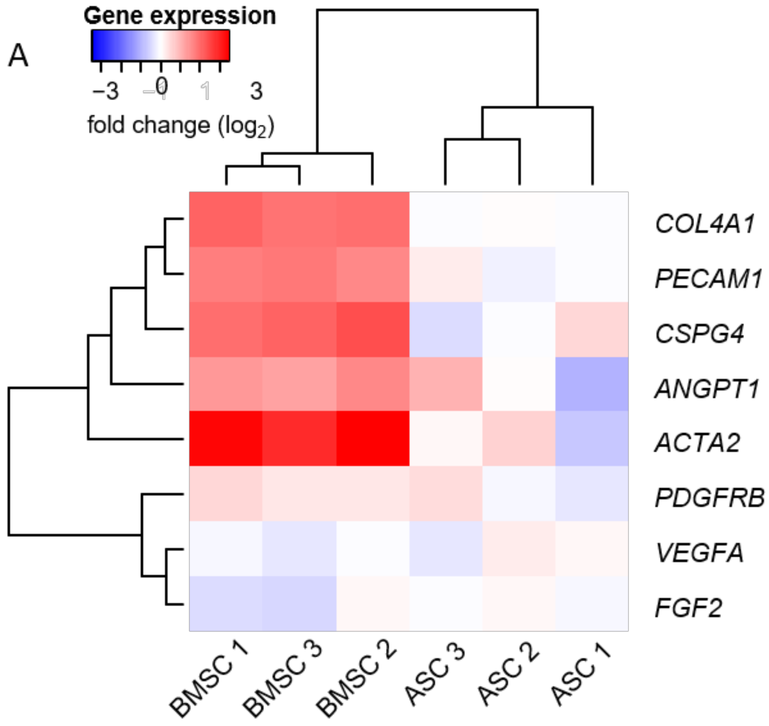

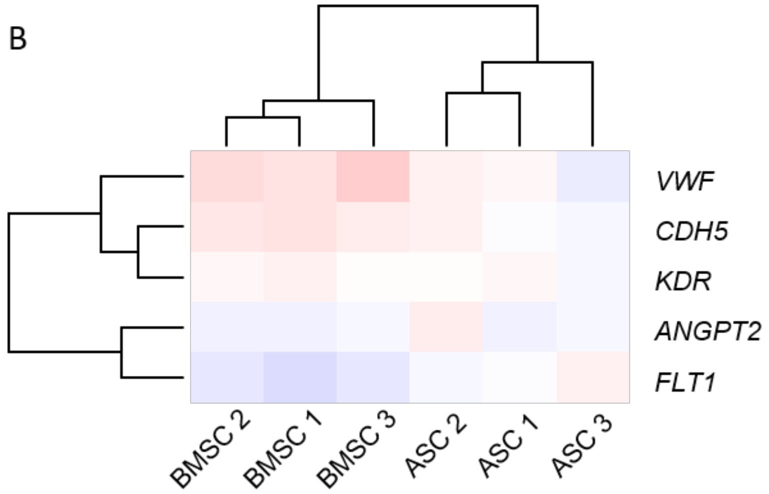

Supplementary Figure 5. Hierarchical clustering shows distinct gene expression patterns in microvascular networks supported by BMSCs and ASCs. Heatmaps showing expression levels of genes normalized to 18S and GAPDH (A) and normalized to PECAM1 (B). Dendrograms produced by hierarchical clustering illustrate the similarity of expression patterns between genes and MSC donor cell lines.

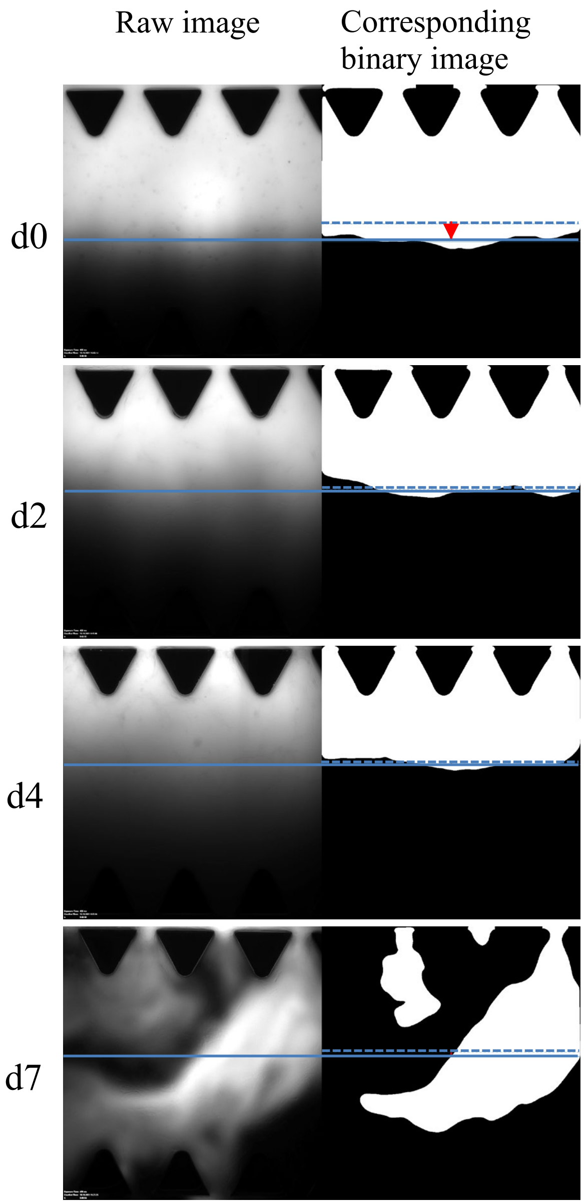

Supplementary Figure 6. Snapshots of image analysis at days 0, 2, 4 and 7. Raw image is shown on left and binarized image for wavefront tracking is shown on right. Blue line represents current estimation of the waveform, dashed line previous waveform location, and red arrow indicates the change between two image indexes. At day 7, the flow condition across the hydrogel is not fully interstitial due to the presence of luminal flow via hollow microvasculature. Thus, the developed waveform tracking method cannot be used for flow rate estimation during luminal flow. Donor cell lines BMSC 1 and ASC 3 were used for generation of data presented in the figure.

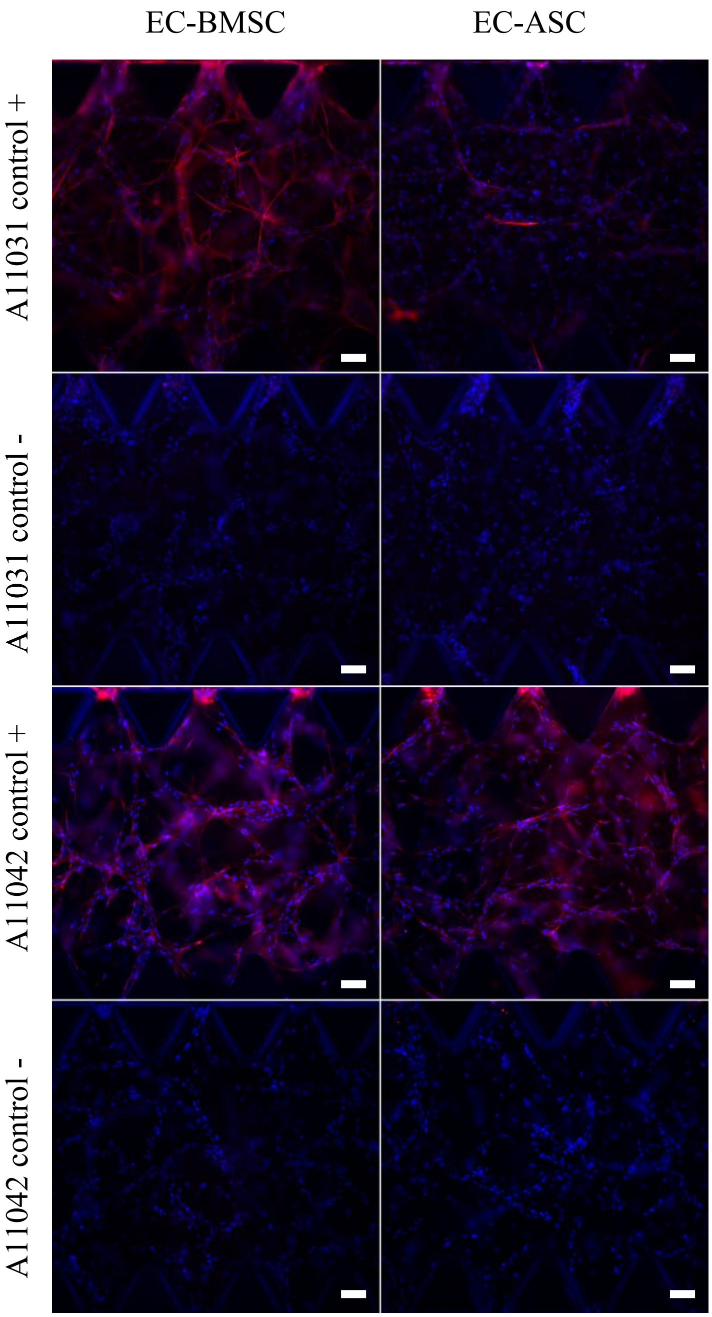

Supplementary Figure 7. Wide-field fluorescence images of positive and negative control for the used Alexa Fluor 568 conjugated secondary antibodies (Thermo Fisher, A11031 and A10042) for immunocytochemical stainings in EC-BMSC and EC-ASC cocultures. Images captured with wide-field fluorescence microscope (Olympus IX 51) with sCMOS camera (Orca Flash4.0LT, Hamamatsu)**.** Nuclei (blue) are stained with DAPI. Scale bars 100µm.

Supplementary videos

EC-BMSC, perfusable

Supplementary Video 1. Microbeads flowing through the microvascular network established by EC-BMSC co-culture. The 2 μm polystyrene microbeads were introduced into one of the media channels after 7 days of culture, entered the microvascular network, flow through, and subsequently exit into the opposite media channel demonstrating formation of a perfusable microvascular network. BMSCs induced formation of perfusable microvascular networks by ECs with multiple entry points upstream of the luminal flow and several exit points downstream of the luminal flow regardless of BMSCs cell line used for co-culture. The video represents data generated using donor cell line BMSC 1.

EC-ASC, partially perfusable

Supplementary Video 2. Microbeads flowing through the microvascular network established by EC-ASC co-culture. The 2 μm polystyrene microbeads were introduced into one media channel after 7 days of culture. The luminal flow was created by imposing a hydrostatic pressure drop between two media channels which allows the microbeads to travel through the microvascular network. Only well interconnected microvascular networks with open lumens were able to carry beads through the networks indicating formation of a perfusable microvascular network. Two ASCs cell lines induced formation of partially perfusable microvascular networks by ECs with a couple entry points upstream of the luminal flow and one exit point downstream of the luminal flow in part of the chips while the third ASCs cell line did not lead to formation of perfusable microvascular networks. The video represents data generated using donor cell line ASC 2.

EC alone, non-perfusable

Supplementary Video 3. Perfusability assessment in ECs cultured alone. The 2 μm polystyrene microbeads were introduced into one media channel after 7 days of culture and the luminal flow was created by imposing a hydrostatic pressure drop between two media channels. Due to absence of open lumens at the hydrogel-media interface in EC alone culture the microbeads were not able to enter the microvascular network but stuck at the hydrogel-media interface. ECs cultured alone did not form perfusable microvascular networks. Scale bar, 1000 μm.
